# Chloroindazole based estrogen receptor β ligands with favorable pharmacokinetics promote functional remyelination and visual recovery

**DOI:** 10.1038/s41598-025-20254-9

**Published:** 2025-10-08

**Authors:** Micah Feri, Sung Hoon Kim, Flavio D. Cardenas, Alyssa M. Anderson, Brandon T. Poole, Devang Deshpande, Shane Desfor, Kelley C. Atkinson, Stephanie R. Peterson, Kendall W. Nettles, Jerome C. Nwachukwu, Moyinoluwa T. Ajayi, Fernando Beltran, David E. Martin, Julio Tapia, Carol D. Curtis, Martin I. Garcia-Castro, Benita S. Katzenellenbogen, John A. Katzenellenbogen, Seema K. Tiwari-Woodruff

**Affiliations:** 1 Division of Biomedical Sciences, School of Medicine at University of California at Riverside, Room 205, 311 School of Medicine Research Building, 900 University Ave, Riverside, CA 92521 USA; 2 Department of Chemistry, and Cancer Center, University of Illinois at Urbana-Champaign, Urbana, IL 61801 USA; 3 Department of Immunology and Microbiology, The Scripps Research Institute, Jupiter, FL 33458 USA; 4 Cadenza Bio, Oklahoma City, Yukon, OK 73120 USA; 5 Departments of Molecular and Integrative Physiology, Cell and Developmental Biology, and Cancer Center, University of Illinois at Urbana-Champaign, Urbana, IL 61801 USA

**Keywords:** Myelin biology and repair, Neuroimmunology, Regeneration and repair in the nervous system, Visual system, Multiple sclerosis, Neurodegeneration

## Abstract

Multiple sclerosis (MS) is a chronic autoimmune, demyelinating, and neurodegenerative disease that results in motor, visual, and cognitive deficits. While existing treatments can slow disease progression, they rarely restore lost neurological function or significantly enhance quality of life. Estrogen receptor β (ERβ) has emerged as a promising therapeutic target due to its ability to activate non-classical signaling pathways involved in neuroprotection, immune modulation, and remyelination. In this study, two chloroindazole-based ERβ-selective ligands, K102 and K110, were identified for their favorable pharmacokinetic profiles and performance in preclinical absorption, distribution, metabolism, and elimination (ADME) screening. These compounds demonstrated biological activity by promoting oligodendrocyte (OL) differentiation in both primary mouse and human OL cultures. In vivo, they enhanced axonal remyelination and improved functional electrophysiological outcomes in two mouse models of MS: experimental autoimmune encephalomyelitis (EAE) and cuprizone diet-induced demyelination. Additionally, K102 and K110 modulated immune responses, supporting OL survival and contributing to motor and visual recovery in EAE mice. These findings provide compelling preclinical evidence for advancing K102 and K110 to clinical development. By simultaneously addressing neurodegeneration and inflammation through ERβ-mediated signaling, these compounds offer a novel and potentially transformative approach to MS therapy.

**Supplementary Information:**

The online version contains supplementary material available at 10.1038/s41598-025-20254-9.

## Introduction

Multiple sclerosis (MS) is an autoimmune disorder characterized by immune system dysregulation, where autoreactive T-cells and B-cells are activated to target and degrade the protective myelin sheath around neurons^1,2^. This damage disrupts normal nerve signal transmission, leading to a range of symptoms that can include motor dysfunction, visual impairments, and cognitive deficits. In the early relapsing–remitting (RR) phase of the disease, symptoms are often reversible. However, over time, RRMS transitions into secondary progressive MS (SPMS), where disease transitions to a progressive worsening of neurological function, leads to significant disability and, in severe cases, may ultimately result in death^1^. Another form of MS is primary progressive (PPMS), where it is characterized by gradual neurological decline from disease onset. Existing treatments for MS can help slow the progression of symptoms and delay the advancement of the disease, but they are largely ineffective in restoring neuronal function or significantly enhancing quality of life^3^. An ideal therapy for MS would need to address multiple aspects of the disease: it should regulate the immune system to prevent harmful demyelination that leads to neuronal loss, while also promoting remyelination of damaged neurons to restore normal function.

Estrogen receptor β (ERβ) is an intriguing target for the development of MS therapies as it is widely expressed in both reproductive and non-reproductive tissues, including the brain. Its broad distribution suggests it plays a key role in regulating inflammation, metabolism, neuroprotection, and tissue maintenance^4,5^. Unlike ERα, which is more dominant in reproductive tissues, ERβ often exerts anti-proliferative and protective effects, making it a target of interest for therapeutic interventions in neurodegenerative diseases^5^. In several in vitro and in vivo studies of MS, the knockdown or knockout of ERβ largely eliminates the beneficial actions of ERβ-selective agonist/ligands, indicating the central role of this ER subtype as a mediator of their efficacy in reversing this disease^6–9^. However, not all ligands with similar ERβ-selective binding affinities have similar beneficial activities, being either inactive^10^ or showing only remyelinating activity, but not both^11^. This indicates that ERβ ligands, like many of those for ERα, are SERMs, i.e., selective ER modulators^12^, where their efficacy is modulated by the response characteristics of the different signal transduction pathways through which they are acting. Past studies have shown that specific indazole-core ligands with strong ERβ binding selectivity^13^ exhibit both immune modulatory and remyelinating properties by acting through distinct signaling pathways separate from classical transcriptional activation^6,11,14,15^. These ligands enhance oligodendrocyte (OL) progenitor cells (OPCs) differentiation to mature OLs and increase remyelination in the cuprizone (CPZ) diet mouse model of MS, and halt disease progression and restore functional activity in the EAE model^11^. Studies were undertaken to further generate novel indazole analogues, characterize and refine the activity profile of these indazoles. In the studies reported here, we have investigated two chloroindazole (IndCl) ligands, K102 and K110, with promising ERβ/ERα binding affinities, immune modulation and myelinating activities, which are the product of extensive profiling (See Discussion), in some cases together with their developmental precursor compound K101^13^.

In this report, comprehensive evaluations of these chloroindazole-derived ERβ ligands were conducted to assess their efficacy, ERβ selectivity, safety profile, and pharmacokinetic properties, including blood–brain barrier penetration—all aspects critical for advancing these compounds toward clinical trials. To enable early and reliable detection of therapeutic effects in such trials, optic neuritis (ON) metrics, a common early manifestation of MS in humans and mice, was used^16^. Non-invasive techniques were prioritized: visual evoked potential (VEP) measured latency changes reflecting demyelination, electroretinograms (ERG) assessed photoreceptor and neuronal dysfunction, optical coherence tomography (OCT) quantified retinal nerve fiber layer thinning^17^, and RNA profiling by NanoString analysis identified inflammatory and remyelination biomarkers. These approaches revealed that ERβ ligands: i) preserved optic nerve integrity by reducing axonal loss and demyelination, ii) modulated cytokine and chemokine levels (e.g., suppressed CXCL10 and TNFα), and iii) accelerated OL maturation via ERβ-dependent signaling. Notably, dual mechanisms—immunomodulation and direct remyelination—were validated as essential for functional recovery in chronic EAE. The combined pharmacokinetic and multimodal efficacy data position these ligands as candidates for clinical evaluation, for both RRMS and PMS to target inflammation and demyelination.

## Materials and methods

*Experimental Animals, Ethical Statement and Euthanasia:* Except when noted otherwise, all experiments were conducted using wildtype C57BL/6J mice (Envigo), in accordance with NIH guidelines for the care and use of animals and study is reported in accordance with ARRIVE guidelines and approved by the IACUC at the University of California, Riverside. Mice were bred and housed in UCR vivarium facilities under a 12-h light/dark cycle with ad libitum access to food and water. At the end of the experiment mice were deeply anesthetized and euthanized with isoflurane.

*ADME Studies, ER Binding and Reporter Gene Assays, and PK Studies:* A series of standard in vitro ADME studies were performed at Pharmaron Ltd. (Shanghai, China). ERα and ERβ ligand binding affinity were done by competitive ligand binding assays using tritiated estradiol as tracer and full length purified human ERα and ERβ (Table 1) and ER reporter gene assays were done in HEK 293 T cells transfected with expression plasmids for ERα and ERβ and an ER-luciferase responsive reporter; both were done in our laboratories as previously described^18,19^. In initial pharmacokinetic (PK) studies done at Pharmaron, CD1 mice received intravenous (IV), subcutaneous (SC), and oral (PO) administration at 3 doses. Pharmacokinetic studies to establish K_p uu_ values were conducted by BioDuro (Shanghai, China), and PK studies done in female C57/BL/6J mice, rats, beagle dogs, and cynomolgus monkeys at oral doses (PO) of 40 mg/kg were done at Pharmaron.

**Table 1:** In Vitro and In Vivo Studies of Chloroindazole ERβ Ligands. Summary of the results of characterizing for K102 and K110 in terms of their the ERβ and ERα binding affinities, their ADME properties (rates of metabolism by human liver microsomes or hepatocytes and their inhibition of various cytochromes P450), their pharmacokinetic profiles, and their efficacy in rates of remyelination in the CPZ model. For details, see Table S1 and Figure S1 and Methods.

Activity	Compound	
	K102	K110
*ER Binding* ^1^		
*RBA β/α* = *ratio*	24.0/0.37 = 65	4.35/0.09 = 48
*ADME (Human)*		
*Microsome stability t*_*1/2*_	87 min	51 min
*Hepatocyte stability t*_*1/2*_	20 min	110 min
*CYP inhibition* ^2^	All ≤ 50% but 3A4T 77%	All < 50% but 1A2 52%
*PK Profile* ^3^		
*Brain/Plasma Ratio(%)*	35 (SC) 40 (PO)	22 (SC) 46 (PO)
*Bioavailability %F*	141 (SC) 40 (PO)	133 (SC) 30 (PO)
*AUCinf plasma/brain* h*ng/mL[or g]	1390/480 (SC)	1403/303 (SC)
	991/391 (PO)	780/354 (PO)
*Brain retention t*_*1/2*_ * h*	2 h (SC) 4.8 h (PO)	1.1 h (SC) 7.4 h (PO)
*K*_*p,uu,brain*_ * Cmax* ^6^	0.63	0.8
*Cuprizone Assay* ^4,5^		
*RM/N*	94% (SC) 76% (PO)	84% (SC) 100% (PO)
*RM/RM-V*	149% (SC) 121% (PO)	133% (SC) 127% (PO)

1 RBA is relative binding affinity (estradiol = 100)

2 CYP inhibition tested at 10 μM against 1A2, 2B6, 2C9, 2C19, 2D6, and 3A4M/T

3 Dosage: IV at 5 mpk, SC at 10 mpk, PO at 25 mpk with CD1 mouse

4 RM/N –remyelination with cpd. vs. normal; RM/RM-V – remyelination with cpd. vs. spontaneous remyelination without cpd

5 Administration route and dosage and: SC administration and 5 mg/kg for each compound

6 Single dose oral PK in mouse at 40 mg/kg

*Mouse and Human primary cell cultures: Primary OPC/OL culture* were isolated from the cortices of postnatal day 0–1 C57BL/6J mice of both sexes, using established protocols^11,20,21^. *Mouse OPC and Neuronal Co-cultures*: Primary neuronal cultures were established in Neurobasal medium with B27 and plated on poly-L-lysine-coated coverslips. After 12 days, OPCs were isolated, counted, and added to neuronal cultures and media was replaced with neuronal and proliferation media for 3 days, then switched to maturation media that maintained both neurons and OLs. *Human iPSC-derived OPCs* (Tempo’s iOligo™) were expanded, cryopreserved, and later plated on Matrigel-coated coverslips. After 3 days in growth medium, cells were treated for 3 weeks with T3 containing maturation medium. Differentiation into myelin-producing OLs was assessed using O4 (OL surface marker on pre-OLs) and myelin basic protein (MBP) immunohistochemistry. In all conditions compounds or vehicle were added to culture during differentiation for either 5 days (mouse) or 18 days (human). Described in detail in the Supplementary Methods.

*Immunocytochemistry and Image Analysis of cell cultures:* After treatment, cells were fixed and stained for MBP and DAPI. Coverslips were imaged using a confocal microscope at 10× and 40× objectives. Cell densities were quantified using ImageJ and normalized to control conditions. MBP-positive cells, their process extensions, and highly branched OLs were counted to assess differentiation. Co-culture images were analyzed for MBP and β3-tubulin colocalization using JACoP^22^ in ImageJ, with results validated by manual quantification. Data was analyzed and visualized using Prism software.

*EAE induction and treatment:* EAE was induced in 8–10-week-old wild-type C57BL/6J mice using MOG₃₅_-_₅₅ peptide^11,14,23–25^. Mice were scored daily using a standard clinical scale (0–5) and tested for their ability to stay on a rotarod^11^. Peak EAE, occurring around days 18–22, represents the acute phase of maximal neurological impairment, when most animals exhibit severe paralysis progressing from the tail and hind limbs to the forelimbs, reflecting extensive demyelination and axonal injury^26^. At peak disease (18–22 days post-immunization), animals were grouped into EAE+Vehicle, EAE+K102, and EAE+K110. Treatments (5 mg/kg/day) were administered via daily subcutaneous injections at peak disease, with dosages based on average body weight and prepared in 10% ethanol/90% miglyol 812N. Data from four EAE experiments are presented in Fig. 3B.

*CPZ administration and treatment:* Mice were fed a diet containing 0.2% bis(cyclohexanone)oxaldihydrazone (CPZ, Teklad Custom Diets, Madison, WI) for 12 weeks, as previously described^15,27^. A subset of mice was perfused immediately following the 12-week CPZ regimen (12wkDM). The remaining mice were returned to a normal diet for 3 weeks to induce remyelination and were treated with either vehicle (3wkRM+V), K102 (5 mg/kg/day; 3wkRM+K102), or K110 (5 mg/kg/day; 3wkRM+K110). These mice were perfused and their brains were processed for IHC.

*Optical coherence tomography, electroretinograms and visual evoked potentials:* OCT was acquired using the spectral domain‐OCT (R2200 840 nm HHP; Leica, Deerfield, IL) at peak EAE disease according to previously published methods^23,28^. Each image was taken 3 times and averaged. Automatic segmentation of retinal layers was performed using Bioptigen Diver 3.0 software (Leica Microsystems, Deerfield, IL). ERG and VEPs were measured using the HMsERG200 (I-VIVO, Henderson, NV) as previously described^23,28^. Animals were dark adapted for a minimum of 5 h prior to recording. During acquisition, animals were anesthetized with continuous 2% isoflurane. Traces were averaged and filtered for 60 Hz noise and a low-pass filter. MATLAB was used to adjust baselines and smoothed prior to measuring ERG and VEP amplitudes and latencies.

*Animal Perfusions, tissue preparation, and IHC:* Mice were deeply anesthetized and euthanized with isoflurane and intracardially perfused with ice‐cold PBS followed by 10% formalin in PBS (Fisher Scientific, Hampton, NH). Eyes, optic nerves, and brain were collected and processed for IHC as previously described^23,28^. Antibody details are listed in Supplementary Table S6.

*Splenocyte Isolation and Cytokine Analysis:* Spleens were collected from mice prior to transcardial perfusion, and splenocytes were isolated, counted, and resuspended in RPMI medium^11^. After 1 h incubation, cells were stimulated with 25 µg/mL MOG₃₅-₅₅, and supernatants were collected 48 h later. Cytokine and chemokine levels in the supernatants, including pro- and anti-inflammatory markers as well as chemokines were analyzed by The Cytokine Core (Indianapolis, IN).

*Imaging and Quantification:* Retina, optic nerve, and brain sections were imaged using an Olympus BX61 spinning disk confocal microscope with 10× and 40× objectives^23,28^. Z-stack images were captured and processed using Slidebook 6 and cellSense software. Immunofluorescence intensity and cell counts were quantified using NIH ImageJ, and statistical analysis was performed with GraphPad Prism.

*RNA Extraction and NanoString nCounter Gene Expression Assay:* RNA was isolated from a single optic nerve of normal, EAE+Vehicle, and EAE+K102 mice^28^. The RNA samples were analyzed using the nCounter Mouse Neuropathology panel (NanoString Technologies) (Supplementary Data Set 1). Fold changes were calculated using normalized data for the three groups (Supplementary Data Set 2).

*Software and URL links used for graphics and data analysis*: Biorender (https://www.biorender.com/); GraphPad Prism 10.6.0 (https://www.graphpad.com/); Slidebook 6.0 (https://www.intelligent-imaging.com/slidebook); cellSense (https://evidentscientific.com/en/products/software/cellsens); nSolver 4.0 (https://nanostring.com/products/ncounter-analysis-system/ncounter-analysis-solutions/).

*Statistics:* Statistical analysis was conducted using GraphPad Prism 6 (for details, SI-Materials). For cell culture experiments, one-way ANOVA followed by Tukey’s post hoc test was used for multiple comparisons, with significance set at α ≤ 0.05. For EAE clinical scores, two-way unbalanced ANOVA with Dunnett’s multiple comparisons test was applied. In IHC studies, three sections per mouse were analyzed for each brain region and tissue type (retina, optic nerve), with 6–8 mice per group. Analyses included data from both eyes and one way ANOVA followed by Fisher ‘s LSD test was performed for ERG, VEP, and OCT. Gene expression was analyzed after calculating log2 fold changes, Welch’s t-test (unequal variance t-test)for group comparisons. Significance levels were defined as *p < 0.05, **p < 0.01, ***p < 0.001, and ****p < 0.0001. Statistical summaries are provided in Supplementary Tables S7-12.

## Results

### In vitro binding, gene activation assays and an ADME screen

Compounds IndCl-o-Me (K102) and IndCl-o-OH (K110) structures are shown in Figure S1. They were selected for this study based on ADME screening results (summarized in Table 1 and Table S1) that included comparisons with the parent compound, IndCl (K101), as well as their high binding affinity and selectivity for ERβ over ERα. The ERβ/ERα relative affinity ratios were 65 for K102^11,11^ and 48 for K110 (Table 1). This ERβ selectivity is further demonstrated in reporter gene assays, where chloroindazoles K101, K102, and K110 were tested alongside ERβ-selective reference compounds—diarylpropionitrile (DPN), ERB041, and LY500307; ERα-selective ligand propylpyrazoletriol (PPT); as well as estradiol (E2) and diethylstilbestrol (DES), which are equally active on ERα and ERβ. The dose–response curves of reporter gene activation are shown in Figure S1, and their potencies (EC_50_ activities) with their Hill coefficients are given in Table S2. The relative ERβ/ERα potencies of all ERβ-selective compounds reflect their ERβ/ERα binding affinities, with relatively minor quantitative differences likely due to the nature of a cell-free competitive binding assay with purified receptors vs. an activity assay done in cells in culture. Many of the ERβ activation curves have Hill coefficients significantly > 1, suggestive of positive cooperativity of ERβ activity; with ERα, most Hill coefficients were distinctly below, a mark of negative cooperativity. This ER subtype-selective differential in cooperativity could also underlie the marked ERβ functional activity we find with the chloroindazoles in the studies herein.

The results of the screen of their metabolic stability in mouse and human liver microsomes and hepatocytes, inhibition of key cytochromes P450, and plasma protein binding are summarized in Table 1, Table S1. Compared to the initial lead compound K101, both K102 and K110 had substantially slower overall metabolism by both human and mouse microsomes and hepatocytes. Notable is the greater stability of K102 in human microsomes and human hepatocytes, and of K110 in human hepatocytes. None of the compounds showed worrisome CYP inhibition, with K102 and K110 having less inhibition of CYP1A2 and CYP3A4T than K101. As uncharged, lipophilic molecules, all these indazole compounds had substantial protein binding in mouse and human plasma, but nothing greater than what is typical for known potent, bioactive ligands for the ERs.

### Pharmacokinetic and brain/plasma distribution studies in mice and comparative clearance rates in mice, rats, dogs, and monkeys

Expanded pharmacokinetic profiling was performed on the two new compounds, K102 and K110. Mice were dosed intravenously at 5 mg/kg, subcutaneously at 10 mg/kg, and orally at 25 mg/kg. Levels of the administered compound were analyzed in plasma and brain over a 24-h period. The kinetic profiles and a summary of the pharmacokinetic parameters are presented in Figures S2 and S3, and Table S3, respectively.

Overall, both K102 and K110 have acceptable PK parameters for further development and are overall relatively similar (Table S3). For both compounds, the clearance rate from blood (IV injection, CI _obs_) is the same; the plasma exposure for both compounds (AUC_0-t_) are somewhat greater for SC than PO dosing, but the clearance from brain (T_1/2_) is slower by PO dosing. Bioavailability (F) for plasma is 30–40% PO and 130–140% SC, with little difference between the two compounds. The brain/plasma exposure ratios K_p_, which reflect the AUC ratios above, are 0.2–0.6 for all dosing, but generally around 0.4, with ratios after PO dosing at the high end (Figures S2B and S3B), indicating clearly that these chloroindazole ERβ ligands are brain penetrant. Since the PK parameters, along with ADME and safety profiles, do not clearly favor one compound over the other, additional factors, such as their relative efficacy in various bioassays (described below) must be considered for compound selection.

The good brain uptake ratio encouraged us to investigate further the unbound or free ligand concentration in plasma vs. brain, K_*p,uu,brain.*_ This pharmacokinetic parameter specifically measures how much drug, not bound to proteins or tissue components, is available for binding to the ERβ target in the brain relative to the blood, and as a more meaningful index of blood brain barrier penetration it is important for developing CNS drugs. The K_*p,uu,brain*_ for K102 and K110 were 0.63 and 0.80, respectively, again indicating effective blood–brain barrier penetration of the unbound compound and hence, a clearer measure of its potential for clinical translation (Table S4). Also, both compounds, but particularly K110 had markedly high K_p uu_ values for the uterus. Under our EAE therapy treatment conditions, however, neither compound caused an increase in uterine weight, which is an ERα-driven response (see below and Figure S6ii). This is consistent with the high ERβ selectivity of their action and lack of activity through ERα.

Lastly, a pharmacokinetic study of K102 clearance was conducted, where it was dosed PO at 40 mg/kg in female C57BL/6 mice, Sprague–Dawley rats, beagle dogs and cynomolgus monkeys and followed over a 24-h period (Table S5 and Figure S4). Rats exhibited the highest exposure, while dogs showed the lowest; exposure levels in mice and monkeys were intermediate and closely aligned. In monkeys—the species most physiologically similar to humans, systemic exposure was sustained with reduced clearance over a 24-h period (Figure S4), further supporting this compound’s potential for clinical translatability as an orally active agent.

### Chloroindazole ERβ ligand treatment increases OL differentiation and axon myelination in vitro

To assess the impact of varying concentrations of K102 and K110 on OL differentiation, primary mouse OPCs were treated with vehicle, and with 10 nM, and 100 nM of K101, K102, and K110 (Supplementary Figure S5i-ii)^11^. Compared to vehicle-treated wells, the number of MBP+ cells significantly increased in a concentration-dependent manner with 10 nM and 100 nM treatments of K101, K102, and K110 (Supplementary Figure S5iii).

**Fig. 1:**
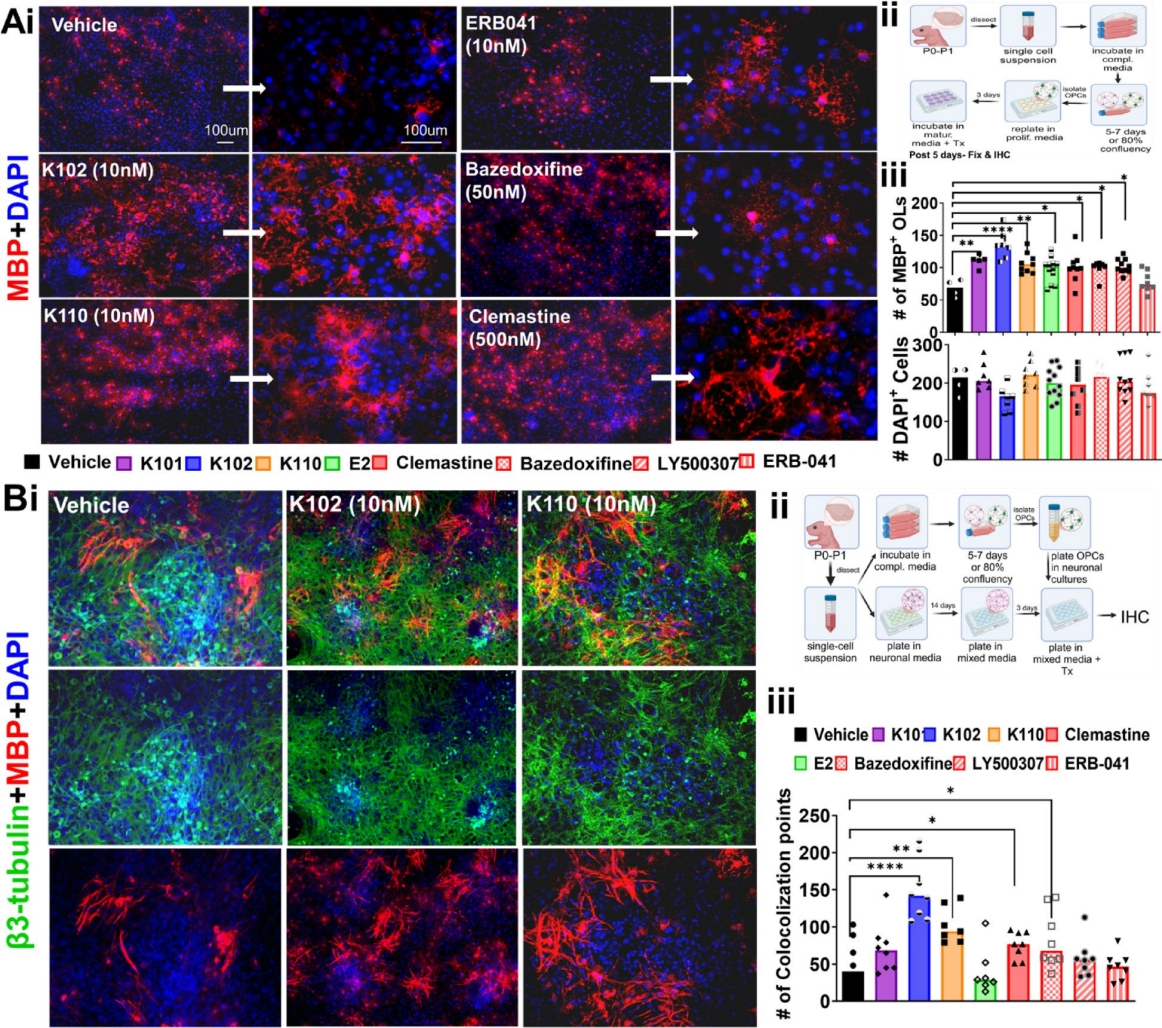
OL differentiation and axon myelination in response to various compounds: (**A**) The extent of OL differentiation was assessed in the presence of 10 nM of K101, K102, and K110, alongside known remyelinating compounds, including ERB-041 (10 nM), clemastine (500 nM), bazedoxifene (500 nM), LY500307 (30 nM), and E2 (50 nM) (Ai-ii). While the total number of cells remained unaffected by treatment, a significant increase in MBP + OLs was observed with K102 and K110 compared to the vehicle control (Aiii). **(B)** The potential of these compounds to promote axon myelination was evaluated in co-cultures of primary cortical neurons and OPCs. Dissociated cortical neurons were maintained for 10 days before the addition of shaken OPCs, which were co-cultured in proliferative OPC + neuronal media for three days. The medium was then switched to OL differentiation media combined with neuronal media for five days, supplemented with either vehicle control, 10 nM of K101, K102, and K110, or known remyelinating compounds, including ERB-041 (10 nM), Clemastine (500 nM), Bazedoxifene (500 nM), LY500307 (30 nM), and E2 (50 nM) (**Bi-ii**). Neuronal cell bodies and axons were labeled using a β3-tubulin antibody, while mature myelinating and non-myelinating OLs were identified as MBP + cells with a highly branched morphology. A significant increase in OL processes wrapping axons was observed in cultures treated with K102, K110, Clemastine, and Bazedoxifene (**Biii**). Figure 1Aii and 1Bii created in BioRender. Feri, M. (2025)

The extent of OL differentiation was compared in the presence of 10 nM of K101, K102, and K110 alongside known remyelinating and ERβ ligand compounds, including ERB-041 (10 nM), Clemastine (500 nM), Bazedoxifene (500 nM), LY500307 (30 nM), and E2 (50 nM) (Fig. 1 Ai-ii)^29^. The total number of cells (assayed with DAPI reactivity) was not affected by the treatment; however, a significant increase in MBP+OLs was observed in K102- and K110-treated wells as compared to the vehicle-treated wells (Fig. 1Aiii).

The ability of compounds to influence axon myelination was investigated in co-cultures of primary cortical neurons and OPCs. Dissociated cortical neuron cultures were maintained for 10 days. On the 10th day, shaken OPCs were added to these neurons and co-cultured in proliferating OPC+neuron culture media for 3 days^30^. The co-cultures were then switched to OL differentiation media combined with neuronal media for 5 days, supplemented with vehicle control, K101, K102, K110 (10 nM), and other known remyelinating compounds, including ERB-041 (10 nM), Clemastine fumarate (500 nM), Bazedoxifene (500 nM), LY500307 (30 nM), and E2 (50 nM) (Fig. 1Bi-ii). Concentrations used for myelination were chosen based on published results. Neuronal cell bodies and axons were labeled using a β3-tubulin antibody, while mature myelinating OLs were identified as MBP+ cells with a highly branched morphology. A significant increase in OL processes wrapping axons was observed in cultures treated with K102, K110, Clemastine, and Bazedoxifene (Fig. 1Bi-iii). Based on their in vitro OL differentiation and axon myelination activity, as compared to known remyelinating agents, K102 and K110 demonstrate strong potential for in vivo remyelination studies.

### Chloroindazole ERβ ligand treatment increases myelination and OL numbers in a CPZ demyelinating model of MS

**Fig. 2:**
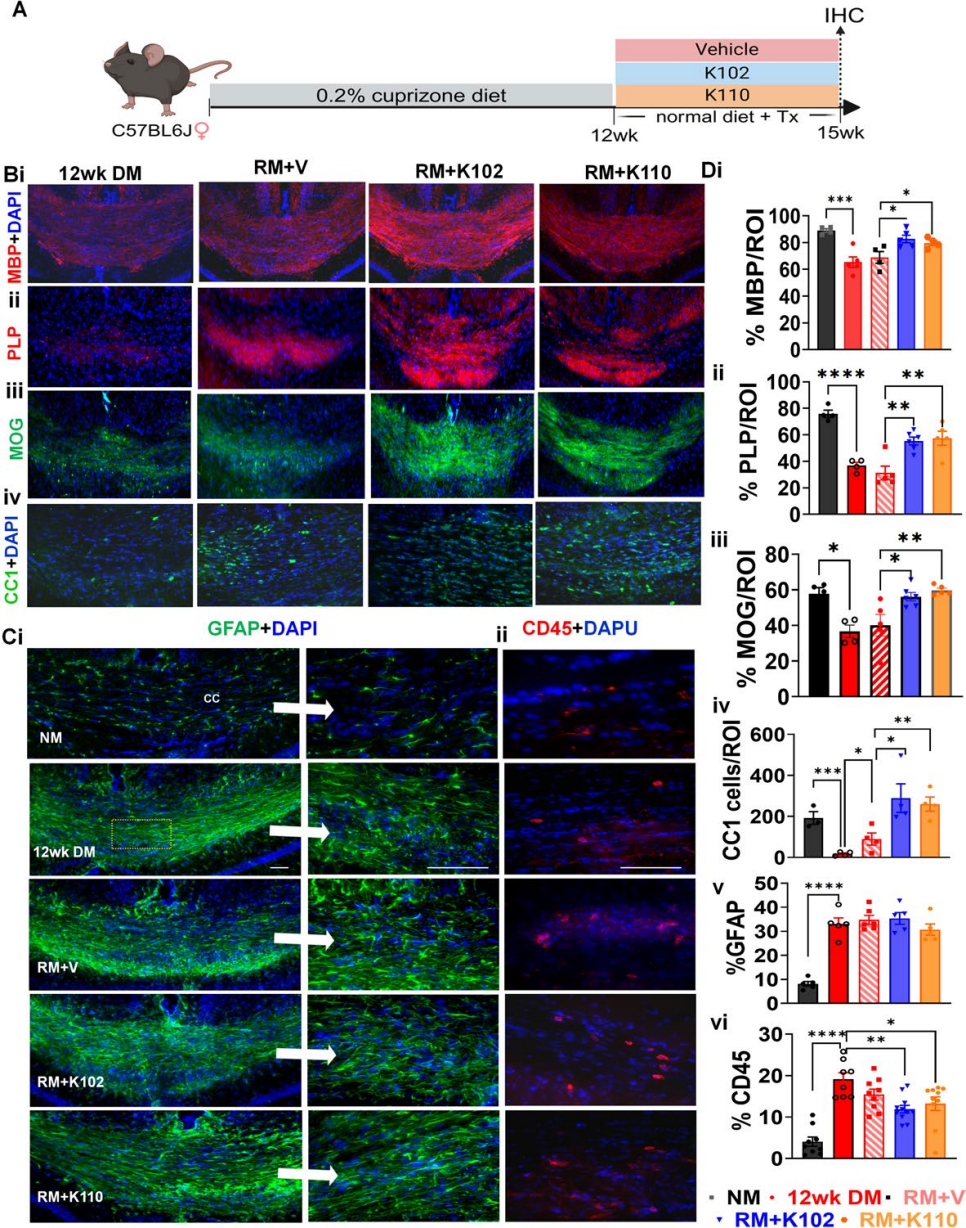
Effects of K102 and K110 on Remyelination: **(A)** The CPZ model was used to assess the direct effects of K102 and K110 on remyelination in the absence of primary infiltrating immune cells. Mice were fed a CPZ diet for 12 weeks (12wkDM) subset of mice were returned to a normal diet to facilitate remyelination and were divided into three treatment groups: RM + V (vehicle), RM + K102, and RM + K110, for three weeks. **(B)** The corpus callosum was analyzed for myelin integrity using MBP, PLP, and MOG staining (**Bi**), the number of mature OLs using anti-CC1 staining (**Bii**), astrocyte activation via GFAP staining (**Ci**, white arrows indicate magnified images from a region denoted in orange dashed box or from other regions on the slide), and inflammation through CD45 staining (**Cii**). Normal control mice exhibited robust MBP, PLP, and MOG presence, a high number of CC1 + OLs, GFAP + astrocytes, and ramified CD45+ microglia. (**C**) After 12 weeks on the CPZ diet, MBP, PLP, and MOG staining intensity and CC1 + OL numbers were significantly reduced, while GFAP + and CD45+ immunoreactivity were markedly increased compared to normal controls (**Bi, Di–iv**). In RM + V mice, myelin intensity remained unchanged; however, CC1 + OL numbers increased compared to 12wkDM mice (**Bi, Di–ii**). **(D**) Treatment with K102 and K110 led to a significant increase in MBP, PLP, and MOG intensity and CC1 + OL numbers compared to RM + V, indicating enhanced remyelination (**Bi, Di–ii**). Additionally, a small but significant decrease in CD45 immunoreactivity was observed in RM + K102 and RM + K110 groups compared to 12wkDM, though GFAP expression remained unchanged (**Ci, Diii–iv**). These findings suggest that K102 and K110 promote remyelination and support OL maturation while modulating inflammatory responses. Treatment with K102 or K110 increased number of mature OLs compared to vehicle. n = 6 mice/group. All graphs represent ± SEM. *p < 0.05, **p < 0.01, ***p < 0.001 using one-tailed unpaired t-test. Figure 2A created in BioRender. Feri, M. (2025) https://BioRender.com/ovc8ubk

To examine the direct effects of K102 and K110 on the extent of remyelination in the absence of primary infiltrating immune response, the CPZ model was used (Fig. 2A). A set of mice were fed for 12 weeks with the CPZ diet (12wkDM), resulting in extensive regional demyelination in the brain, including the corpus callosum (Fig. 2Bi). A subset of mice was assessed at 12wkDM, and the rest of the mice were put back on a normal diet to initiate remyelination and divided into 3 groups for treatment: RM+V, RM+K102, and RM+K110 for 3 weeks. The corpus callosum was assessed for myelin (myelin basic protein-MBP, proteolipid protein-PLP, and myelin oligodendrocyte glycoprotein-MOG, Fig. 2Bi-iii, Di-iii), the number of mature OLs (anti-adenomatous polyposis coli clone-CC1, Fig. 2Biv), astrocyte activation (glial fibrillary acidic protein-GFAP, Fig. 2Ci) and inflammation (leukocyte antigen marker-CD45, Fig. 2Cii). Normal controls displayed robust myelin as seen by MBP, PLP, and MOG staining, many CC1+OLs, GFAP+ astrocytes and ramified CD45+ microglia (Fig. 2B and C).

After 12wkDM, there was a significant reduction in MBP, PLP, and MOG staining density and CC1+OL numbers (Fig. 2Bi-iii and iv) and a significant increase in GFAP + and CD45+ immunoreactivity as compared to normal controls (Fig. 2 Ci-ii, 2Di-iv). Corpus callosum of RM + V mice showed minimal differences in MBP, PLP, or MOG intensity; however, an increase in CC1 + OLs as compared to 12wkDM mice was observed (Fig. 2B, D).Treatment with K102 and K110 displayed a significant increase in both myelin (MBP, PLP, and MOG staining) and a further increase in CC1 + OLs compared to RM + V (Fig. 2B, D). Interestingly, a small but significant decrease in CD45 immunoreactivity but not GFAP was observed in RM + K102 and RM + K110 groups as compared to 12wkDM groups (Fig. 2 Ci-ii, Diii-iv).

These findings suggest that both K102 and K110 promote remyelination in the CPZ model by enhancing myelin recovery and increasing the number of mature OLs. The observed reduction in CD45+ immune cell infiltration further indicates a potential modulatory effect on inflammation. However, the persistence of GFAP + astrocyte activation suggests that while these compounds aid in remyelination, they do not significantly alter astrocytic responses. Overall, these results highlight the potential of K102 and K110 as promising therapeutic candidates for enhancing remyelination in demyelinating conditions.

### Chloroindazole ERβ ligand treatment attenuates EAE clinical disease severity, improves motor performance without increasing uterine weight

**Fig. 3:**
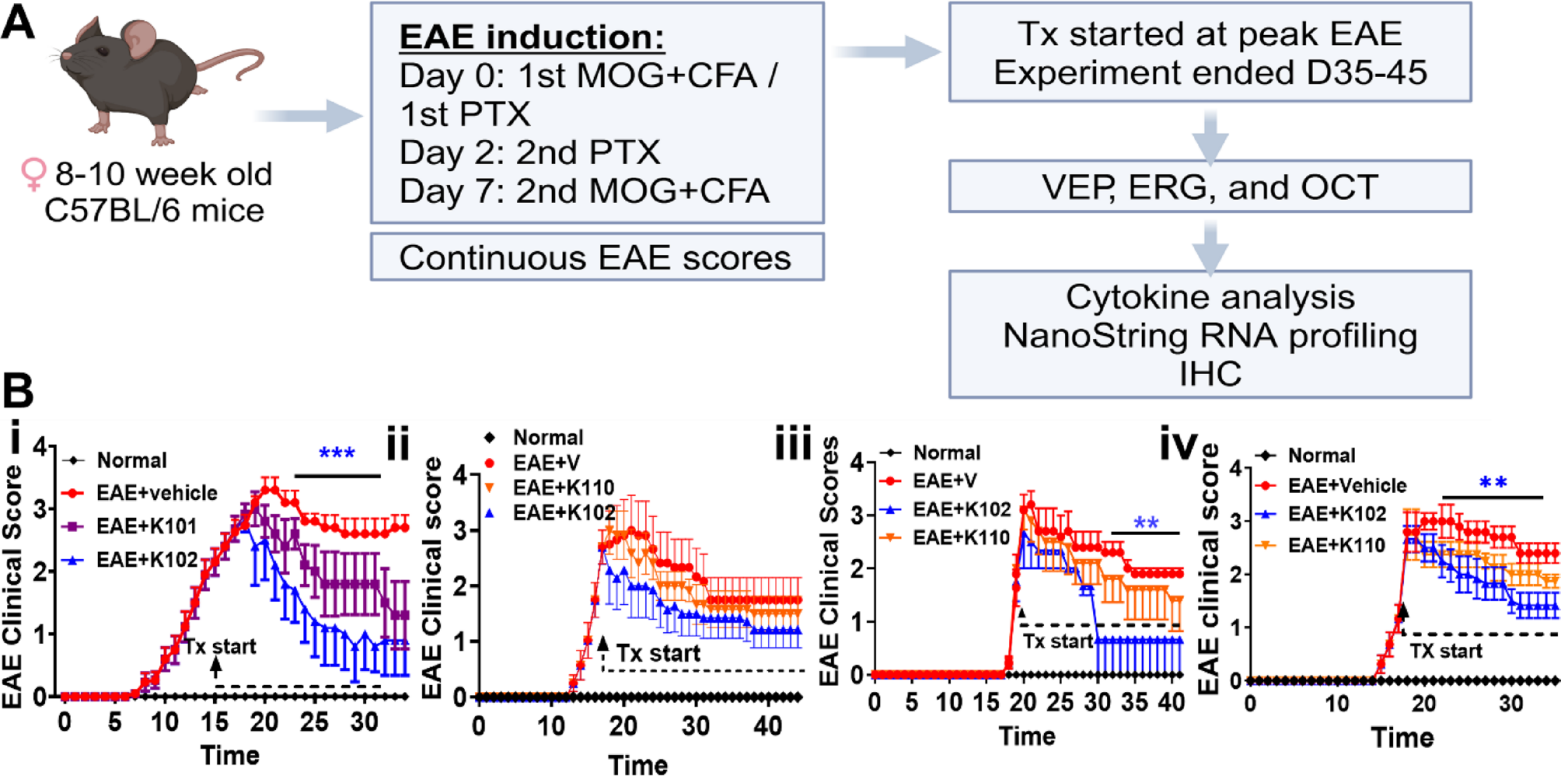
Experimental design for treatment effects in EAE. (**A**) Experimental plan. (**B**) Four separate EAE experiments are shown C57BL/6 J female mice were induced using our 2 MOG induction protocol **(Bi,iii-iv)** or the Hooke laboratories protocol (**Bii,** Kit EK-0111, Inc. Lawrence, MA). Following emulsion injection, mice were injected with pertussis (PTX). Mice were scored for their clinical EAE score daily. EAE mice (red) show signs of disease starting at days 8–17. At peak disease between the days 15–21, therapeutic treatment started and continued daily until the end of the experiment (D35-45). Mice were dosed SC with vehicle (red), K101 (5 mg/kg; purple), K102 (5 mg/kg; blue), or K110 (5 mg/kg; orange). (**Bi**) K102 had a greater therapeutic effect than K101. (**Bii-iv**) Treatment with K102 significantly attenuated clinical disease severity compared to vehicle treatment. K110 had a modest therapeutic effect without reaching significance. N = 6–8 mice/group. Two-Way unbalanced ANOVA with Dunnett’s multiple comparisons test was performed. Mice selected from these 4 EAE assays were also used for VEP, ERG, and OCT to measure visual function, and for cytokine and NanoString gene analyses with results shown in subsequent figures. Figure 3A created in BioRender. Feri, M. (2025) https://BioRender.com/0tod0po

Therapeutic treatment with our initial ERβ ligand K101 has been shown to improve visual pathway dysfunction^23^, motor dysfunction^15^, improve remyelination in the spinal cord, corpus callosum, optic nerve, and modulate the immune system in EAE mice^11,14^. To evaluate the effects of K102 or K110 on EAE clinical disease, C57BL/6 J female mice were induced with EAE following an established protocol^25^ (Fig. 3A). Starting at peak EAE clinical disease (Day 18–22) groups of mice were dosed with vehicle (EAE + V), K102 (EAE + K102), K110 (EAE + K110) and in some experiments K101 (EAE + K101) (Fig. 3A). Treatment with K101 has consistently demonstrated a decrease in EAE clinical disease in our published and current experiment (Fig. 3Bi), whereas treatment with K102 exhibited superior attenuation of EAE clinical disease as compared to K101 in a head-to-head comparison (Fig. 3Bi, iv) and also shown in Karim et al., 2019{Karim, 2019 #128,257}. Interestingly, treatment with K110 did not significantly reduce clinical disease (Fig. 3Bii-iv). In addition to EAE clinical scores, another motor test—the ability to remain on the rotarod—was performed with K102^11,15^. Decreased clinical scores were directly correlated with increased time on the rotarod. The rotarod test, shown in Figure S6i, demonstrated that normal mice were able to stay on the rotarod for the full 300 s. In contrast, EAE mice treated with the vehicle exhibited significant difficulty during peak clinical disease and beyond. Treatment with K102 and K110 resulted in non-significant improvement in the ability of these mice to remain on the rotarod (Figure S6i).

In rodents, exposure to estrogens that specifically target ERα, rather than ERβ, has been associated with increased uterine weight, myometrial thickening, and uterine dilation^31^. Previous studies have reported an increase in post-perfusion uterine weight relative to body weight in mice treated with estriol, estradiol, and ERα agonists^32^. However, no increase in uterine weight relative to body weight was observed in either normal or EAE intact female mice treated with vehicle, K102, or K110 (Figure S6ii, Table S13)^11,14^. The results emphasize the ability of these compounds to alleviate EAE clinical symptoms without stimulating ERα-related effects.

### Chloroindazole ERβ ligand treatment modifies peripheral cytokine and chemokine responses in EAE

**Fig. 4:**
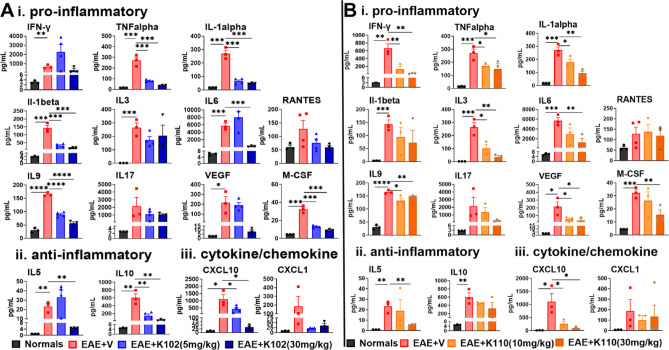
Therapeutic treatment with ERβ ligands modulates peripheral immune response. Splenocytes from K102- and K110-treated mice were collected and stimulated with MOG_35–55_. Supernatants were subjected to cytokine/chemokine analysis. Vehicle-treated mice exhibited significantly increased levels of pro-inflammatory and anti-inflammatory cytokines compared to normal controls. Treatment with K102 (**Ai**) or K110 (**Bi**) significantly decreased pro-inflammatory cytokines compared to vehicle. Treatment with K102 (**Aii**) or K110 (**Bii**) shows a significant decrease in anti-inflammatory cytokines compared to vehicle. Vehicle-treated mice exhibited significantly elevated levels of CXCL10 compared to normal controls. Treatment with K102 (**Aiii**) or K110 (**Biii**) decrease CXCL10 levels compared to vehicle. Production of CXCL1 does not change. n = 3–4 mice/group, One-Way ANOVA with Dunnett’s Multiple Comparisons Analysis.

In MS and EAE, peripherally activated immune cells release inflammatory cytokines and chemokines, triggering demyelination and axonal damage. To examine how different concentrations of K102 (5 mg/kg or 30 mg/kg) (Fig. 4A) and K110 (10 mg/kg or 30 mg/kg) (Fig. 4B) influence the peripheral immune response, splenocytes were isolated from EAE mice at 40 dpi. These splenocytes were then stimulated ex vivo with MOG_35-55_ and analyzed for cytokine and chemokine expression.

Consistent with our previous publications, splenocytes isolated from EAE + V mice exhibited increased production of pro-inflammatory cytokines (e.g., IFNγ, TNFα, IL-1α, IL-1β) (Fig. 4Ai, 4Bi)^11^. Treatment with K102 and K110 reduced pro-inflammatory cytokine levels compared to the vehicle (Fig. 4Ai, 4Bi). Additionally, splenocytes from EAE + V mice showed elevated anti-inflammatory (IL5 and IL10) cytokine production (Fig. 4A and Bii-iiii). However, treatment with either K102 or K110 resulted in significantly lower levels of certain anti-inflammatory cytokines compared to the vehicle (Fig. 4Aiii, 4Biii).

CXCL1 and CXCL10 are leukocyte chemoattractants with opposing effects on OPC survival. CXCL1 plays a crucial role in homeostatic white matter development, OPC proliferation, and survival, whereas CXCL10 promotes OPC death. Splenocytes from EAE + V mice exhibited increased CXCL10 production compared to those from healthy controls (Fig. 4Aiii, Biii). Treatment with K102 or K110 reduced CXCL10 levels compared to vehicle-treated splenocytes, potentially enhancing OPC survival and maturation into myelinating OLs (Fig. 4Aiii, Biii). However, in contrast with the previous findings, current treatment with K102 or K110 did not affect CXCL1 production. These findings demonstrate the capacity of K102 and K110 to modulate the peripheral immune response, highlighting their potential therapeutic impact through their net positive effects.

### Therapeutic treatment with Chloroindazole ERβ ligand, K102 alleviates EAE-induced functional deficits.

**Fig. 5:**
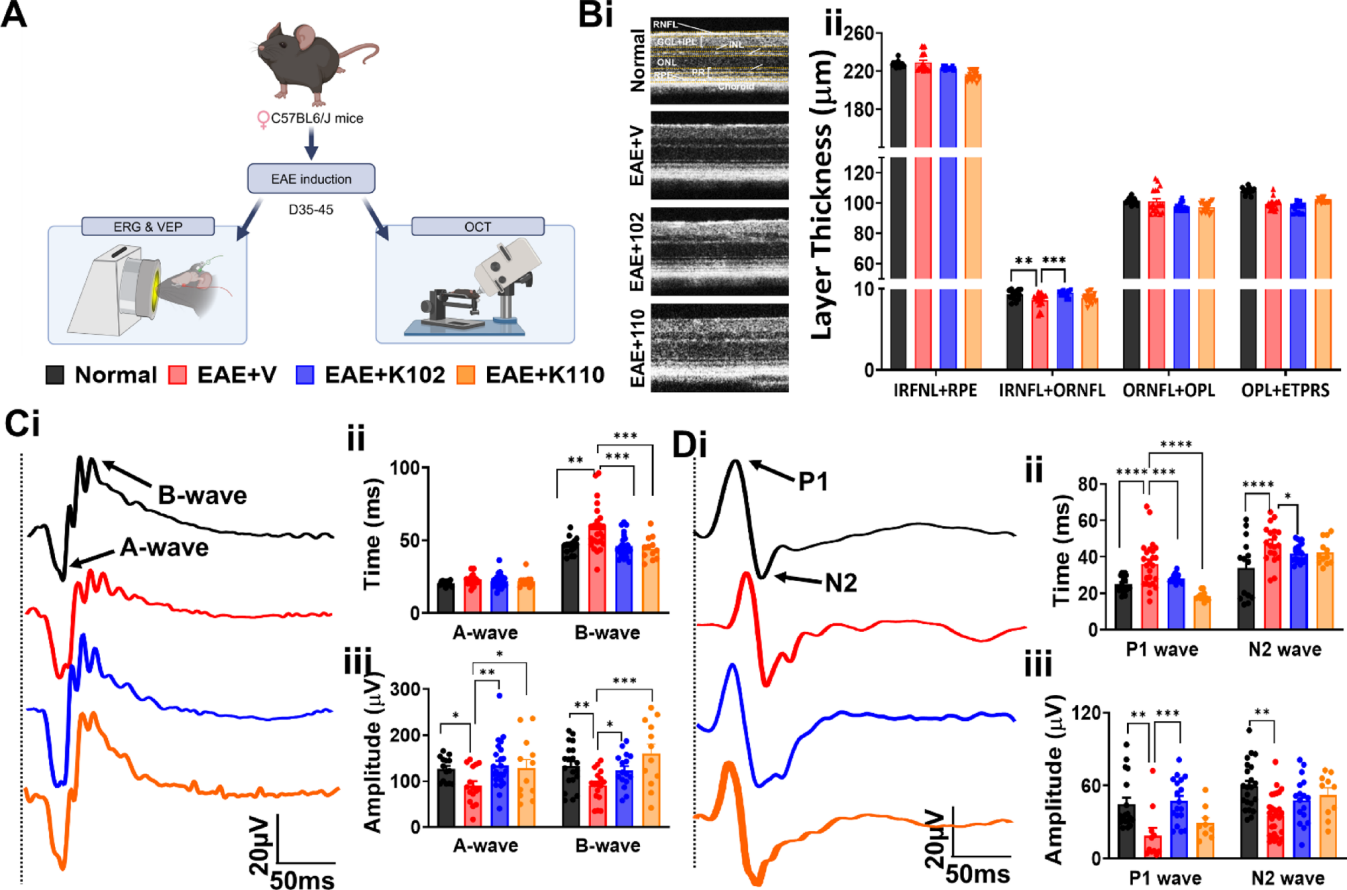
Functional outcomes with ERβ ligand treatment. (**A**) Visual outcomes were measured during chronic disease using OCT, ERG, and VEP. (**Bi)** Representative images of an OCT scan from a normal EAE + V mouse, and from EAE + K102, and EAE + K110 mice. (**Bii**) Quantification of layer thicknesses show EAE + V mice show a significant decrease in RNFL thickness. Treatment with K102 shows an increase in RNFL thickness compared to vehicle. (**Ci**) Representative ERG responses from all treatment groups are shown with A and B wave components demarcated with black arrows on the normal waveform (black). (**Cii**) EAE mice show an increase in ERG B wave latency compared to normal. Treatment with K102 or K110 decreased B-wave latency compared to vehicle. (**Ciii**) A decrease in ERG amplitude was observed in EAE mice. Treatment with K102 or K110 increased ERG amplitude compared to vehicle. (**D**) Visual function was assessed by VEP from normal, vehicle, K102, and K110 mice. (**Di**) Representative VEP responses from all treatment groups are shown with P1 and N2 components defined with black arrows on the normal waveform (black). (**Dii**) EAE mice have an increased VEP latency compared to normal. Treatment with K102 or K110 decreases P1 latency compared to vehicle. (**Diii**) A decrease in VEP amplitude was seen in EAE mice compared to normal. Treatment with K102 increased P1 wave amplitude compared to vehicle. n = 5–8 mice/group. All graphs represent, mean ± SEM. *p < 0.05, **p < 0.01, ***p < 0.001, ****p < 0.0001 level. Figure 5A created in BioRender. Feri, M. (2025) https://BioRender.com/2i536sw

MS has a profound impact on the visual pathway, leading to structural and functional changes^16^. These alterations can be assessed using imaging techniques like OCT and electrophysiological tests such as ERG and VEP, which offer non-invasive insights into the relationship between inflammation, demyelination, and axonal damage in vivo. Consequently, the visual pathway serves as a valuable model for tracking disease progression and assessing new therapies in clinical trials^33^. Our previous work demonstrated the susceptibility of the visual system to inflammation-induced demyelination throughout the course of EAE disease^23,28^. To determine if K102 or K110 improves EAE-induced functional deficits, mice were assessed by OCT, ERG, and VEP at chronic disease (Fig. 5A).

Consistent with our earlier findings, OCT imaging of EAE retinas revealed retinal nerve fiber layer (RNFL) thinning, a hallmark also observed in MS patients^34^ (Fig. 5A, Bi, ii). Treatment with K102, but not K110, prevented the significant reduction in RNFL thickness seen in EAE + V mice (Fig. 5A, Bi, ii), suggesting halted progression or recovery from neuroretinal atrophy.

Functional changes in different retinal layers can be assessed using ERG, which provides insight into retinal function by measuring electrical responses to visual stimuli. ERGs were performed on dark-adapted, anesthetized mice. Normal mice displayed a typical ERG response with an average amplitude of the downward A-wave at 125 µV followed by the upward B-wave with an average amplitude of 125 µV followed by a return to baseline (Fig. 5Ci). However, EAE mice treated with vehicle displayed a significant decrease in A-wave and B-wave amplitude with an increase in A-wave and B-wave latency compared to normal controls (Fig. 5Cii). EAE mice treated with either K102 or K110 revealed a significant increase in both A-wave and B-wave amplitude as compared to vehicle-treated mice. In addition, treatment of EAE mice with K102 or K110 improved B-wave latency that had similar values as a control group (Fig. 5Ciii).

VEPs can serve as markers of demyelination or remyelination, as indicated by lag or decrement in latency. To assess how ongoing treatment with K102 or K110 during EAE influences visual function, VEPs were recorded and compared across normal mice and treated EAE mice. Normal mice exhibited a characteristic robust VEP response, with an average P1 wave amplitude (upward deflection) of 43 µV, followed by an N2 wave amplitude (downward deflection) of 59 µV (Fig. 5Di-iii). In contrast, vehicle-treated EAE mice displayed a significant reduction in both P1 and N2 amplitudes. Additionally, both P1 and N2 peaks shifted to the right, indicating slowed conduction. Measurements revealed an increase in VEP latency, suggesting potential demyelination of the visual pathway in EAE mice as compared to normal mice.

VEP measurements in EAE mice treated with K102 showed a significant increase in peak P1 amplitude and a reduction in P1 latency compared to vehicle-treated EAE mice (Fig. 5Di-iii). In contrast, K110 treatment did not affect P1 or N2 amplitude but did reduce P1 latency (Fig. 5Di-iii).

By incorporating functional outcomes relevant to MS clinical trials, these findings underscore the potential of K102 and K110 in improving visual function, as demonstrated by the recovery of RNFL layer thickness (only in K102) and enhancements in ERG and VEP waveforms.

### Chloroindazole ERβ ligand treatment decreases RGC loss, improves axon myelination, and partially protects against axon damage in EAE optic nerves.

**Fig. 6:**
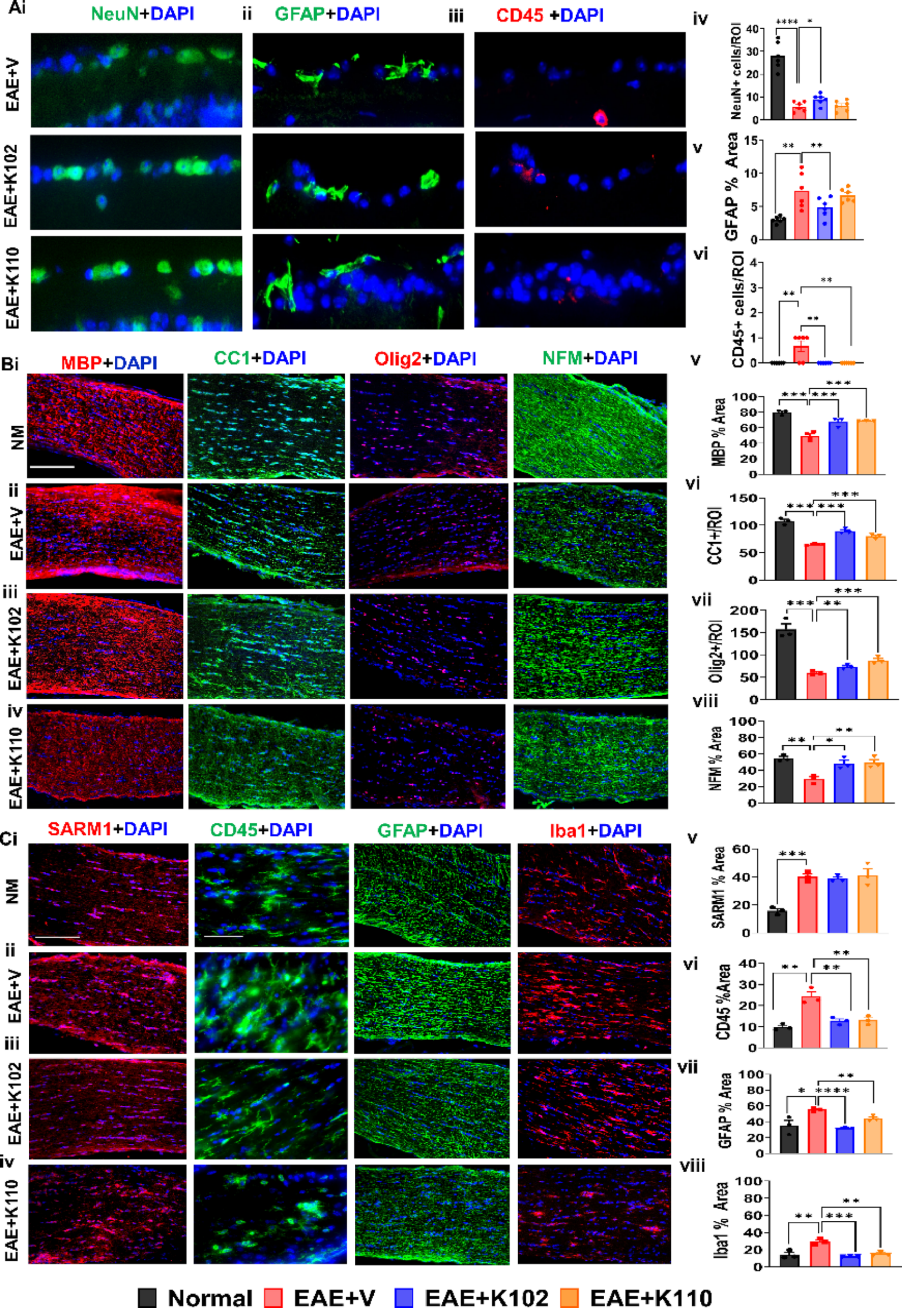
Retinal ganglion cell (RGC) survival, optic nerve myelination, optic nerve axon health and inflammation is modified with K102 and K110. (**A**) Representative images of retinal section shown for EAE + V, EAE + K102, and EAE + K110 treated groups. To evaluate RGC loss, astrogliosis, and immune cell infiltration, NeuN, GFAP, and CD45 immunoreactivity was used. EAE + V mice showed decreased NeuN numbers compared to normal (**Ai, iv**). Treatment with K102 showed an increase in NeuN + cells. To assess astrogliosis and immune infiltration, GFAP and CD45 immunoreactivity were used. EAE + V mice exhibit increased GFAP and CD45 immunoreactivity compared to normal (**Aii-iii, v-vi**). Treatment with K102 exhibited a decrease in GFAP and CD45 while K110 only decreased CD45 immunoreactivity compared to EAE + V mice. (**B**) Representative images of longitudinal optic nerves are shown for NM, EAE + V, EAE + K102, and EAE + K110 treated groups. To evaluate axon myelination, OL lineage and mature OLs, MBP, Olig2, and CC1 immunoreactivity was assessed EAE + V mice decreased myelin intensity, number of OL lineage cells and mature OL numbers compared to normal (**Bi-ii, v-vii**). Treatment with K102 or K110 showed increased myelination, OL lineage cells and mature OL numbers compared to EAE + V mice (**iii-vii**). To assess axon health in the optic nerves, neurofilament (NFM) immunoreactivity was used. EAE + V mice exhibit a significant reduction in NFM immunoreactivity compared to normal (**Bii, viii**). Treatment with K102 or K110 increases NFM staining compared to vehicle (**Biii-iv, viii**). To assess whether SARM1 expression in the optic nerve changes with K102 or K110 treatment, optic nerve sections were stained for SARM1 expression. EAE + V mice displayed a significant increase in SARM1 expression compared to normal (**Ci-ii, v**). K102 and K110 treatment caused no change in SARM1 expression relative to vehicle (**Ciii-v**). To assess K102 or K110 treatment effects leukocyte infiltration into the optic nerve, sections were immunostained with a pan-leukocyte marker, CD45 (**C**).Vehicle-treated optic nerves show an increase in CD45+ infiltration compared to normal controls (**Ci-ii, vi**). Treatment with K102 or K110 exhibited reduced CD45+ infiltration compared to vehicle (**Ciii-iv, vi**). To assess astrocytes and microglia/macrophages during chronic disease, optic nerve sections were immunostained for GFAP and Iba1. EAE + V mice exhibited increased GFAP and Iba1 expression compared to controls, (**Ci-ii, vii-viii**). Treatment with K102 or K110 reduced GFAP and Iba1 staining compared to vehicle (**Ciii-iv, vii-viii**). n = 5–8 mice/group. Scale bar for B i-iii = 100 µm and B iv, v = 10 µm. All graphs represent mean ± SEM. *p < 0.05, **p < 0.01, ***p < 0.001, ***p < 0.0001 level.

**Fig. 7:**
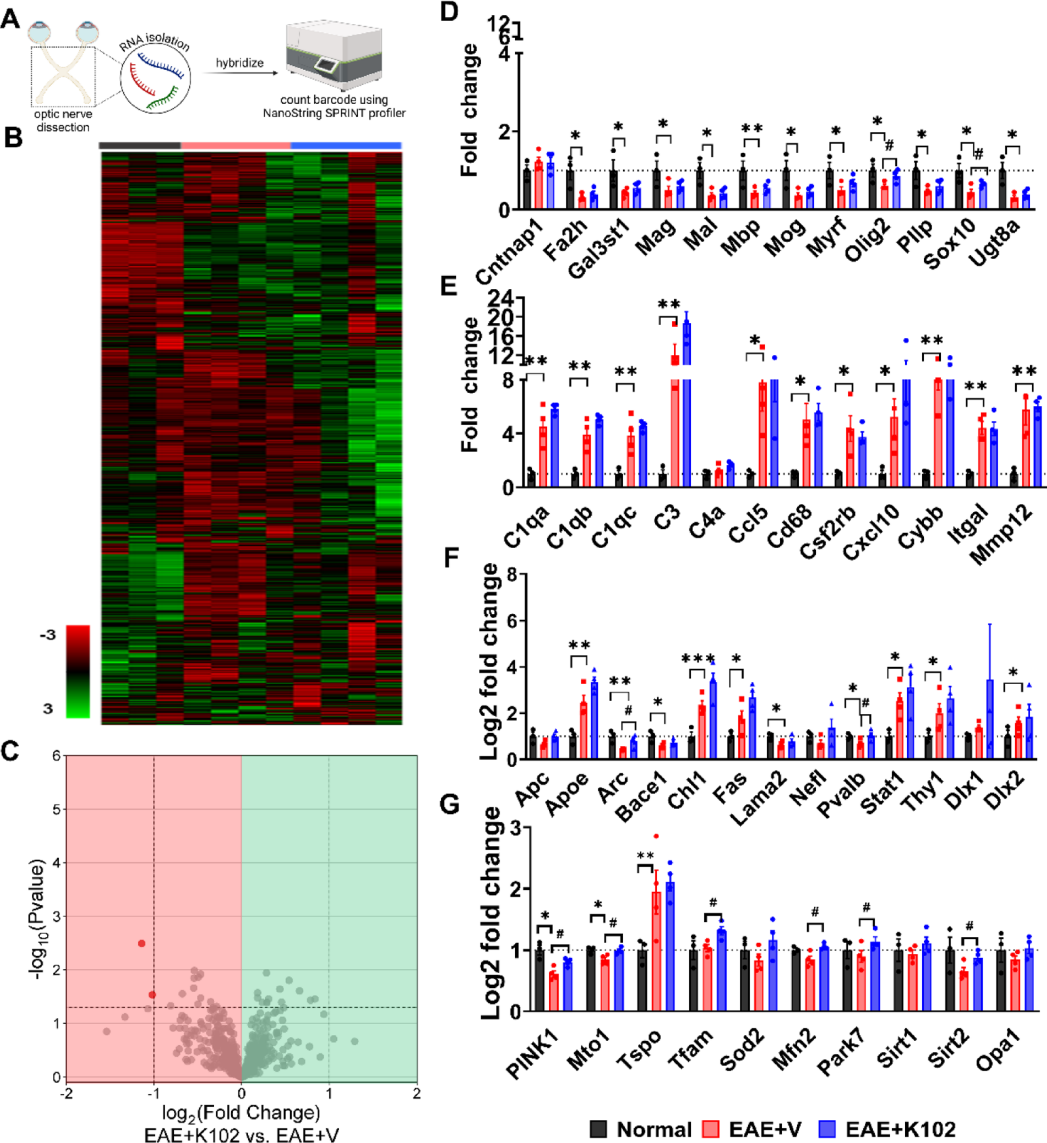
Gene changes with ERβ ligand treatment: **(A)** To assess the differences in gene transcripts during late EAE, NanoString gene analysis using the Neuropathology panel on normal, EAE + V, and EAE + K102 optic nerves. **(B)** Heat map of all genes from the Neuropathology panels. Gene expressions are depicted from low expression (red) to high expression (green). Heat map generated from normalized gene expression data from normal (gray), EAE + V (red), and EAE + K102 (blue) optic nerves using nSolver software. **(C)** Volcano plot showing 2 out of 770 genes are differentially expressed using predefined cutoffs (p < 0.05 with a log2 fold-change in the same direction). (D) Fold change for myelination genes normalized gene counts. Selected genes (*Fa2h, Gal3st1, Mag, Mal, Mbp, Mog, Myrf, Olig2, Pllp, Sox10, and Ugt8a*) show significant downregulation compared to normal optic nerves. Treatment with K102 increases *Sox10* and *Olig2* compared to vehicle. **(E)** Fold change for inflammation genes using normalized gene counts. Selected genes *C1qa*, *C1qb, C1qc, Ccl1, Cd68, Csf2rb, Cxcl10, Cybb, Itgal*, and *Mmp12* show significant upregulation compared to normal optic nerves with no changes observed with K102 treatment. (**F)** Fold change for neuronal health genes from normalized gene counts. Genes such as *Apoe, Chl1, Fas, and Thy1* show significant upregulation (green) while other genes like *Arc, Lama2, Bace1, Pvalb, and* show significant downregulation (red) compared to normal optic nerves. Treatment with K102 upregulates *Arc* and *Pvalb* genes denoting some neuroprotection. **(G)** Fold change for mitochondrial genes using normalized genes counts. Genes such as *Pink1* show an upregulation compared to normal. *Tspo* shows a downregulation compared to normal. K102 treatment shows an upregulation (*Pink1*, *Tfam*, *Mfn2*, *Park7*, and *Srt2*) compared to normal. All graphs represent mean + SEM. *p < 0.05, **p < 0.01, ***p < 0.001, ****p < 0.0001 by one-tailed student’s t-test. Normal and EAE + V data previously published in^28^. Figure 7A created in BioRender. Feri, M. (2025) https://BioRender.com/1z1pvt1

Our current results demonstrate a significant decrease in the RNFL layer in EAE mice, similar to our previously published results^23,28^. RGC degeneration and pronounced GFAP immunoreactivity is evident in both MS and EAE^23,28,35,36^. To quantify the number of RGCs, neuronal nuclear marker (NeuN) was used. Consistent with previous findings, vehicle-treated EAE mice exhibited a significant decrease in NeuN + RGCs compared to normal mice (Fig. 7Ai,iv). When treated with K102, the number of NeuN + RGCs were increased (Fig. 6Ai,iv). To assess astrogliosis and inflammation, retina sections were immunostained with GFAP and CD45. Compared to normal sections, vehicle-treated EAE mice exhibit increased GFAP immunoreactivity and CD45+ cells (Fig. 6Aii-iii, v-vi). In K102 treated mice, there was a significant reduction in both GFAP and CD45 compared to vehicle-treated EAE mice (Fig. 6Aii-iii, v-vi). Treatment with K110 only reduced CD45+ cells compared to vehicle-treated EAE mice.

A key characteristic of MS and EAE-induced pathology is a substantial decrease in axon myelination and a reduction in OL lineage cells^37^. Axon myelination levels and OL lineage cell populations, including OPCs and mature OLs, were quantified. IHC was performed, and levels of MBP immunoreactivity and the number of OL transcription factor 2 (Olig2 +) and CC1 + mature OLs in optic nerves were quantified. Consistent with previous findings, vehicle-treated EAE mice exhibited significantly reduced myelin staining intensity and fewer OL lineage cells, including fewer mature OLs, compared to normal controls (Fig. 6Bi-ii, v-vii). In contrast, EAE mice treated with K102 or K110 showed a significant increase in myelin staining intensity, OL lineage cell numbers, and mature OL counts compared to vehicle-treated EAE mice (Fig. 6Biii-vii).

Another hallmark of MS and EAE is significant axonal damage in white matter regions, including the optic nerve^34,37^. Neurofilaments (NFM), a type of intermediate filament protein, provide structural support and stability to axons. To assess axonal integrity in the optic nerves of EAE mice, NFM immunoreactivity was compared across experimental groups. In normal animals, NFM staining exhibited a robust and coherent pattern, which was significantly diminished in EAE mice treated with vehicle (Fig. 6Bii, viii). However, treatment with either K102 or K110 increased NFM staining intensity and partially restored the organized axonal structure in the optic nerve compared to vehicle-treated EAE mice (Fig. 6Biii-iv, viii).

Axonal damage in neurodegenerative diseases, including MS, involves multiple mechanisms, one of which is the upregulation of sterile alpha TIR motif-containing protein 1 (SARM1), an essential mediator of axon degeneration^38^. To determine whether SARM1 expression in the optic nerve is altered by K102 or K110 treatment, optic nerve sections were immunostained for SARM1. Compared to healthy optic nerves, EAE mice treated with vehicle exhibited a significant increase in SARM1 expression (Fig. 6 Ci-ii, v). Notably, treatment with K102 or K110 did not reduce SARM1 expression compared to vehicle-treated EAE mice, indicating that SARM1 remains involved in axonal damage despite treatment (Fig. 6Ciii-v).

These findings demonstrate that K102 and K110 treatment promote remyelination in EAE by enhancing myelin recovery and increasing OL numbers. Additionally, K102 and K110 restore neurofilament integrity without altering SARM1 expression. Overall, these results underscore the therapeutic potential of K102 and K110 in promoting axonal remyelination in a model of inflammatory demyelination.

### Chloroindazole ERβ ligand-treated optic nerves reveal decreased immune cell infiltration.

Consistent with our previous studies, therapeutic treatment with K101 has been shown to reduce leukocyte infiltration and astrocyte activation^11,14^. To evaluate the effects of K102 and K110 on leukocyte infiltration into the optic nerve, tissue sections were immunostained with the pan-leukocyte marker CD45 (Fig. 6C). Optic nerves from vehicle-treated mice exhibited increased CD45+ infiltration compared to normal controls (Fig. 6 Ci-ii,vi). However, treatment with K102 or K110 resulted in a reduction in CD45+ infiltration relative to the vehicle-treated group (Fig. 6Ciii-iv, vi).

To further assess astrocyte and microglia/macrophage activity during chronic disease, optic nerve sections were immunostained for GFAP and ionized calcium-binding adaptor molecule 1 (Iba1) (Fig. 6C). EAE + V mice showed increased GFAP and Iba1 immunoreactivity compared to controls, indicating astrogliosis and activated microglia/macrophages (Fig. 6 Ci-ii,vii-viii). In contrast, treatment with K102 or K110 reduced GFAP and Iba1 staining compared to vehicle-treated mice (Fig. 6Ciii-iv, vii-viii).

The observed decrease in CD45+ immune cell infiltration, along with the reduction in GFAP and Iba1 staining following K102 or K110 treatment, suggests a modulatory effect on inflammation.

### Chloroindazole ERβ ligand treatment increases myelin and protects against axon damage in EAE optic tract.

A significant increase in inflammation, and a decrease in axon myelination along with axon damage, is observed in the optic tracts of EAE mice^23,28^. To determine whether K102 exhibits similar remyelinating and immunomodulatory effects, brain sections containing the optic tract were stained for MBP, Iba1, and GFAP (Figure S7). EAE + V mice displayed a significant decrease in MBP expression (Figure S7Ai, iv), a significant increase in Iba1 (Figure S7Aii, v), and GFAP (Figure S7Aiii, vi) compared to normal controls. Treatment with K102 alleviated EAE-induced demyelination relative to vehicle-treated mice. Additionally, K102 treatment significantly reduced Iba1 and GFAP immunoreactivity, indicating decreased astrocyte and microglia activation. These findings suggest that K102 increases expression of myelin-associated markers while modulating the immune response.

Beyond myelination, axonal integrity was also assessed in the optic tract of K102-treated mice (Figure S7Bi, iii). EAE + V mice exhibited reduced NFM expression compared to normal mice, whereas K102 treatment significantly increased NFM levels relative to vehicle-treated mice (Figure S7Bi, iii). Furthermore, SARM1, a key regulator of axonal degeneration, was evaluated (Figure S7Bii, iv). While EAE + V mice showed a significant increase in SARM1 immunoreactivity compared to normal mice, K102 treatment did not produce a significant change relative to vehicle treatment. These results suggest that K102 contributes to overall axonal protection without modifying the SARM1 pathway.

### Chloroindazole ERβ ligand treatment modulates RNA transcripts during chronic EAE.

Genes associated with myelination, inflammation, and axon health are altered in EAE optic nerves compared to normal optic nerves^28^. To examine how K102 treatment affects these gene transcripts, RNA from treated optic nerves was analyzed using the NanoString Neuropathology gene expression panel (Fig. 7A, Supplementary Data Set 1). Among the 770 genes assessed (Fig. 7B, Supplementary Data Set 2), two—*Ccr2* and *Cpt1b*—were significantly altered in K102-treated optic nerves compared to vehicle-treated optic nerves (Fig. 7C). *Ccr2* is a chemokine receptor involved in recruiting immune cells to sites of inflammation. *Cpt1b* (carnitine palmitoyltransferase 1B) is an enzyme critical for fatty acid oxidation.

Previously we have shown that myelination genes such as *Sox10, Olig2, PIIp, Gal3st*1, *Mbp, Mal, Mog, Ugt8a*, and *Fa2h* are significantly downregulated in EAE optic nerves compared to normal optic nerves^28^. Additionally, we show other myelination genes such as *Mag*, and *Myrf* are significantly downregulated in EAE optic nerves compared to normal (Fig. 7D). K102 treatment led to an increase in two genes essential for OL differentiation and maturation: *Sox10 and Olig2* in optic nerves relative to vehicle-treated mice (Fig. 7D). However, K102 did not significantly impact the EAE-induced increase^28^ in selected inflammation-related complement system genes such as *C1qa, C1qb, C1qc, and C3* (Fig. 7E). Additional genes such as *Ccl5, Cd68, Csf2rb, Cxcl10, Cybb, Itgal*, and *Mmp12* were not significantly impacted with K102 treatment relative to vehicle treated EAE mice. Genes related to axon health such as *Apoe*, *Chl1*, *Fas*, and *Thy1* were seen to be upregulated in EAE while *Arc*, *Lama2*, *Bace1*, and *Pvalb*, were downregulated^28^. Treatment with K102 showed changes in *Arc* activity-regulated cytoskeletal gene in the brain encodes a protein essential for learning, memory, and synaptic plasticity by regulating the strength and function of neuronal connections) and *Pvalb* (parvalbumin important in GABAergic neuronal activity) increased compared to vehicle treated optic nerves (Fig. 7F). Given that mitochondrial dynamics are known to be disrupted in EAE, we also examined mitochondrial gene expression in treated and untreated optic nerves. In EAE optic nerves, *Tspo* was upregulated compared to normal controls (Fig. 7G). Notably, K102 treatment resulted in a significant increase in mitochondrial-related genes, including *Pink1, Tfam, Mfn2, Park7, and Sirt2,* compared to untreated EAE optic nerves (Fig. 7G). Enhanced expression of *Tspo* or mitochondrial translocator protein, *Pink1* or PTEN-induced kinase (a serine/threonine kinase), and *Mfn2* or mitofusin 2 (crucial for maintaining the shape and structure in mitochondria), *Sirt2*, an NAD + (nicotinamide adenine dinucleotide)-dependent deacetylase all have role in maintaining and protecting mitochondria during disease^39^.

These findings indicate that K102 facilitates remyelination through the transcriptional upregulation of genes associated with OL differentiation and myelin synthesis, modulation of neuroinflammatory and axonal maintenance pathways, and enhancement of mitochondrial biogenesis and function.

### Chloroindazole ERβ ligand treatment of human OLs increases OL differentiation.

**Fig. 8:**
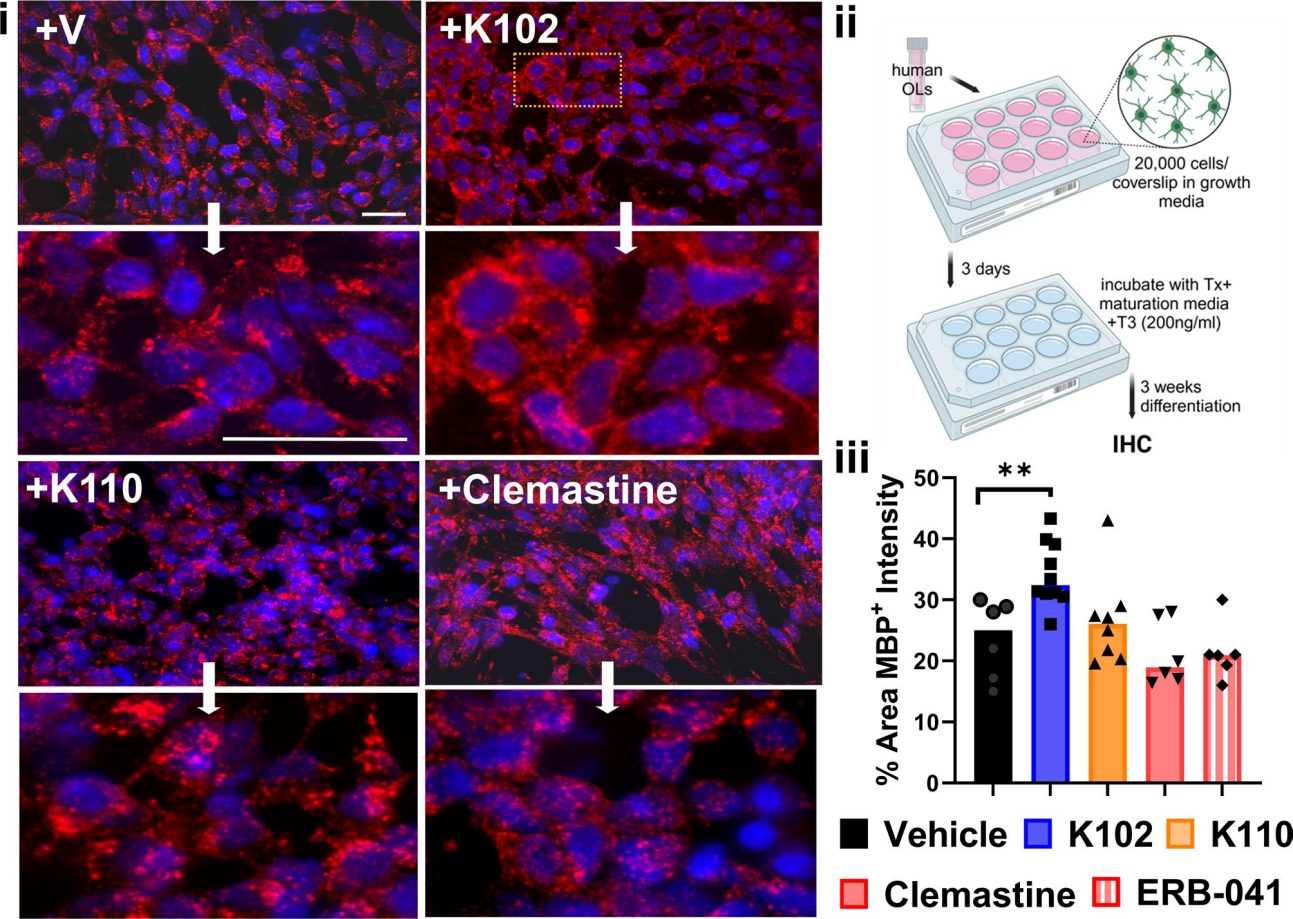
K102 increases OL differentiation in human OLs: (i) Representative confocal fluorescent images were acquired using a 20X objective, with selected regions (indicated by white arrows) further imaged at higher resolution using a 40X oil objective. Experimental conditions included Tempo’s iOligo-derived OPCs/OLs cultured in maturation media alone (vehicle) or treated with K102 (10 nM), K110 (10 nM), Clemastine (400 nM), or ERB-041 (10 nM). OLs were immunostained for myelin basic protein (MBP, green) and counterstained with DAPI (blue). (ii) Cells were thawed and plated on Matrigel coated coverslips and kept in Growth Medium for three days. Cells were then treated with remyelinating drugs (K102, K110, ERB-041, Clemastine fumarate) in Maturation Media complemented with T3 [200 ng/mL]. Media was switched every two days. After 18 days, cells were fixed with 10% formalin and stained. (iii) Effects of treatment on the intensity of MBP + OLs were quantified. Analogue K102 showed a significant increase in the intensity of MBP + OLs compared to vehicle-treated cells. No significant differences in total number of cells were observed between groups. There were 3 wells/treatment groups. Data was analyzed using One-Way ANOVA with Dunnett’s multiple comparisons test. Among the tested compounds, only K102 significantly enhanced MBP levels compared to the vehicle control (**p < 0.005), suggesting its potential role in promoting human OL differentiation and axon myelination (iii). Scale bars in + V panels are 100 µM.

Tempo’s iOligo (OPC) cells form branched bipolar and tripolar structures when cultured as a monolayer and express OL markers, including O4, NG2, and CNPase. Under T3 induction, iOligo™ further expresses MBP, a marker of mature OLs. The ability of human OL lineage cells to differentiate in response to K102, K110, Clemastine, as a positive remyelination control and ERB-041 (ERβ selective agent) were compared to a vehicle control (Fig. 8i-ii). Immediately after thawing, Tempo’s iOligo cells were plated on Matrigel-coated six-well plates, passaged upon reaching confluence, and stored for later use. For differentiation experiments, cells were initially plated on poly-L-ornithine (PLO)-coated six-well plates and compared to Matrigel-coated plates. Although cells on PLO remained healthy, they formed multilayered clusters (2–4 cell layers thick) even in the presence of T3 and maturation medium (Figure S8). Since the cells grew more uniformly in monolayers and exhibited better process formation, Matrigel was chosen for subsequent differentiation experiments. After plating human OPCs and allowing two days of growth in expansion media, the culture medium was switched to T3 + maturation medium and evaluated for MBP expression (Fig. 8i). Cells in all conditions showed robust differentiation with MBP immunoreactivity. However, only K102 significantly increased MBP levels as compared to the vehicle control (**p < 0.005), highlighting its potential role in promoting human OL differentiation and axon myelination (Fig. 8i-iii).

## Discussion

**Fig. 9:**
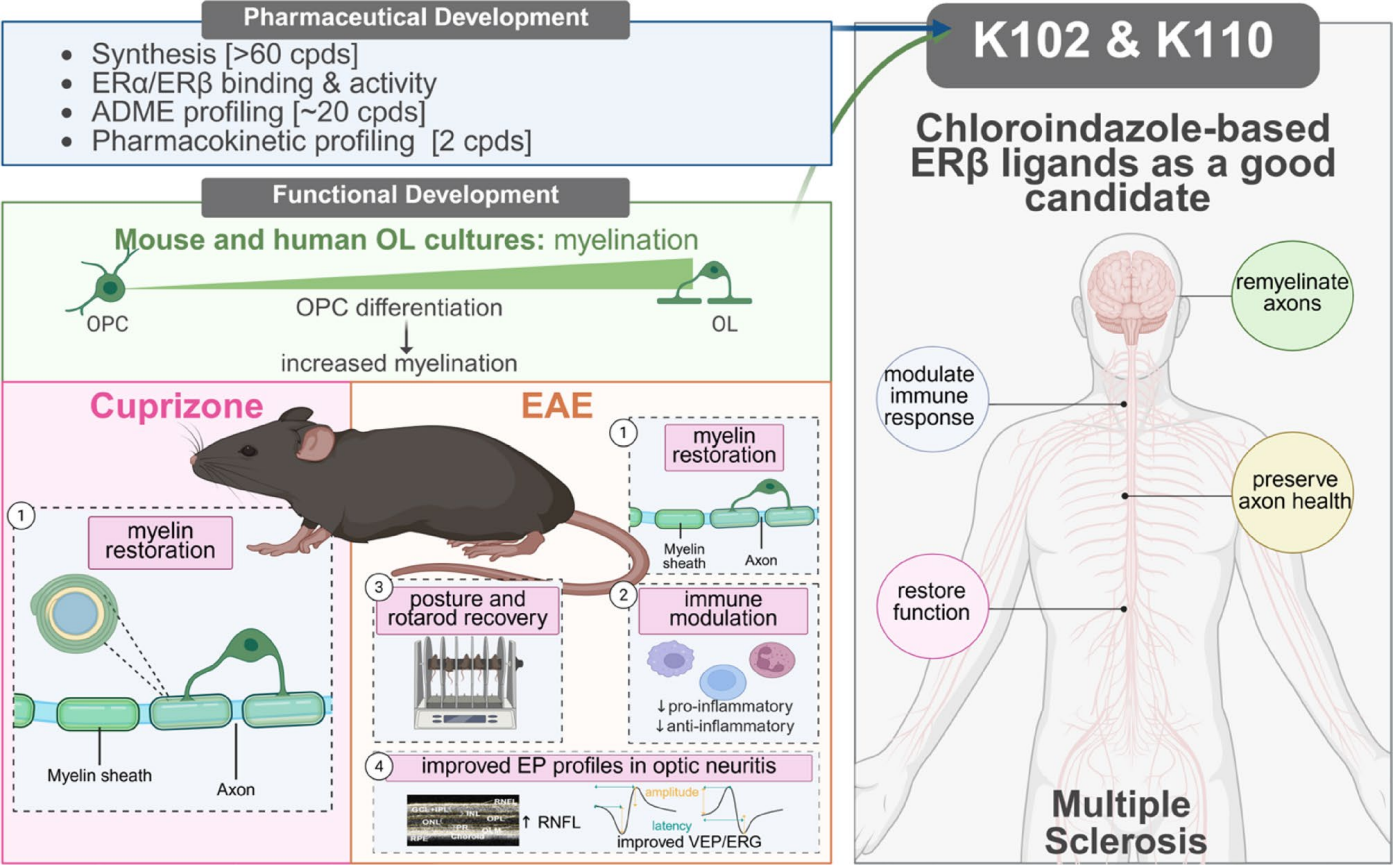
Graphical Abstract of ER β ligand, IndCl analogues remyelinating activity. Graphical representation of the primary findings. Two chloroindazole-based ERβ-selective ligands, K102 and K110, were identified for pharmaceutical development by their synthesis, enhanced ER binding and activity, ASME profiling, and pharmacokinetic profiling. For functional development, these compounds demonstrated increased myelination by OPC differentiation into OLs. In the cuprizone-induced demyelination model, treatment with K102 and K110 1) restored myelin. In the experimental autoimmune encephalomyelitis (EAE) model, K102 and K110 1) restored myelin, 2) modulated the immune system, 3) recovered mouse posture and rotarod performance, and 4) improved evoked potential profiles in optic neuritis. These findings provide compelling preclinical evidence for advancing K102 and K110 to clinical development by addressing remyelination, immune modulation, and preserving axon health leading to overall functional restoration. Figure 9 created in BioRender. Feri, M. (2025) https://BioRender.com/a3yagm.

In this study, we demonstrate that small-molecule chloroindazole ERβ ligands, optimized for selective ERβ engagement and favorable pharmaceutical and pharmacokinetic profiles suitable for oral administration, exert multiple therapeutic effects in mouse models of multiple sclerosis (Fig. 9). In both the EAE and CPZ models, these compounds enhance remyelination and modulate the immune milieu, resulting in improved clinical outcomes and neuronal function, without inducing uterine hypertrophy, an indicator of ERα activation. These findings significantly extend our prior work and represent a critical step toward achieving Investigational New Drug (IND) status. Furthermore, we characterize the pharmacokinetic properties of the chloroindazoles in rodents, dogs, and non-human primates, including brain penetration and free drug concentrations, and define a panel of non-invasive blood and neuronal biomarkers that can be employed for monitoring therapeutic responses in both preclinical and clinical settings.

It is important to interpret our findings within the broader context of ongoing research into the development of MS therapeutics in both preclinical models and human studies, particularly regarding the therapeutic potential of ERβ activation. Current disease-modifying therapies largely focus on suppressing inflammatory processes and have been effective in reducing relapse rates in RRMS; however, they provide limited benefit in promoting remyelination or preventing disease progression, especially in progressive forms of MS. Thus, there remains a critical unmet need for therapies that not only modulate immune responses but also enhance endogenous repair mechanisms, including remyelination and neuroprotection.

*Recent clinical trials of MS therapeutics.* Recent clinical trials were focused on promoting remyelination in MS and have explored diverse mechanisms to stimulate OPC differentiation and myelin repair. Opicinumab (Anti-LINGO-1 Monoclonal Antibody) targets LINGO-1, a protein that inhibits OPC differentiation. Despite promising preclinical results, the SYNERGY Phase II trial (NCT01864148) did not meet its primary endpoint, leading to discontinuation of further development^40^. Originally an antihistamine, clemastine fumarate was identified for its potential to promote remyelination through muscarinic receptor antagonism. The ReBUILD Phase II trial (NCT02040298) demonstrated a modest reduction in VEP latency with clemastine, suggesting some remyelinating activity^41^. However, clemastine accelerates accumulation of disability in PMS by enhancing pyroptosis, and the trial was halted^42^. Commonly used for type 2 diabetes, metformin has shown potential in enhancing OPC differentiation and remyelination in preclinical studies. Clinical trials are ongoing to assess its efficacy in MS, both as monotherapy (NCT05298670) and in combination with clemastine (NCT05893225)^43^. A synthetic thyroid hormone, liothyronine (L-T3) has been investigated for its role in promoting OPC differentiation^44^. While early-phase trials (NCT03098433) indicated potential benefits, concerns about cardiovascular toxicity have limited its progression in clinical development. Thus, a major unmet clinical need in MS, the lack of effective therapeutic options for progressive disease remains.

*Estrogens as MS therapeutics.* Estrogens have demonstrated significant effects on OL survival and axon myelination. E2 promotes OPC differentiation and OL maturation by acting through ERα and ERβ, as well as its membrane-bound receptor GPR30, all of which are expressed by OLs^45–47^. Preclinical studies indicate that prophylactic treatment with estrogens exerts notable immune and neuroprotective effects in mouse models of MS^15,24,48^. Estriol, a pregnancy-related estrogen hormone, has been investigated in clinical studies for its potential therapeutic effects in MS^49^. A Phase 2 trial combining oral estriol with glatiramer acetate (GA) showed a 32% reduction in annualized relapse rate compared to placebo^50^. The treatment’s efficacy may stem from its combined anti-inflammatory and neuroprotective effects, particularly in enhancing cognitive function. Notably, estriol has not been tested as a monotherapy; its value appears to lie in augmenting GA’s outcomes, especially concerning cognitive preservation and cortical protection.

Recently, bazedoxifene/BZA, a selective ER modulator with higher binding affinity for ERα than ERβ, was identified through an unbiased high-throughput screen for remyelinating activity. Its efficacy was subsequently validated in preclinical models. BZA has existing safety data in humans, having been assessed in the Selective Estrogens, Menopause, and Response to Therapy (SMART) trials^51–53^. It is currently being tested for remyelination potential in a double-blind, randomized, controlled, delayed-start Phase 2 clinical trial (NCT04002934).

In spite of somewhat positive results with estriol and GA treatment, estrogen therapies have been regarded with cautious optimism due to their feminizing and cancer causing effects, which mostly arises due to its binding to ERα.

*ERβ as a promising target for MS therapeutics.* ERs, particularly ERα and ERβ orchestrate multifaceted signal transduction pathways that are both spatially and temporally regulated within cells^54,55^. While ERα predominantly mediates estrogenic activity in reproductive tissues and is chiefly expressed in the hypothalamic and limbic regions of the CNS, ERβ exhibits higher expression levels in the prefrontal cortex, hippocampus, and cerebellum^56^. This distribution suggests that ERβ plays a pivotal role in cognitive functions, synaptic plasticity, neuroprotection, and remyelination. Upon ligand binding, ERβ can initiate cellular responses through several mechanisms. Notably, direct genomic action by binding to estrogen response elements in DNA to regulate gene transcription; indirect genomic action by tethering with other transcription factors to modulate gene expression; and non-genomic action by activating kinase signaling cascades from membrane or other or extranuclear sites. Hence, while ligand binding to ERβ is essential for initiating its effects, this interaction alone does not predict its biological efficacy.

Studies involving BERKO (ERβ knockout) mice have highlighted the critical role of ERβ in CNS function^57^. These mice exhibit defective OL differentiation in the primary motor cortex, leading to abnormalities in myelin sheath formation. Additionally, they display increased locomotor activity coupled with impaired motor coordination, likely attributable to disrupted cortical myelination and synaptic function. These findings underscore the importance of ERβ in maintaining normal myelination and cognitive function, highlighting its potential as a therapeutic target in CNS disorders.

ERβ ligands have been extensively studied for their potential neuroprotective and remyelinating effects in MS models. ERβ selective compounds such as DPN, genistein, AC-186, 4-[2-phenyl-5,7-bis(trifluoromethyl)-3-pyrazolo[1,5-a]pyrimidinyl]phenol (PHTPP), 3β-androstenediol (Adiol), Δ^5^-3β-Adiol, and IndCl have demonstrated varying degrees of efficacy in promoting remyelination and neuroprotection in different experimental settings^58^. Among different ERβ ligands, ERB-041 has progressed to clinical trials for inflammatory diseases. Although it did not demonstrate efficacy in rheumatoid arthritis, it was deemed safe in human trials^59^. This safety profile paves the way for further exploration of ERβ ligands in clinical settings for remyelination therapies. For instance, DPN has been shown to activate PI3K/Akt/mTOR signaling pathways in OLs, leading to enhanced remyelination in mouse models of MS^60^.

Despite their high affinity and selectivity for ERβ, these ligands do not uniformly produce favorable immunomodulatory or remyelinating outcomes. This variability in results can be attributed to differences in study designs, including the use of gonadectomized animals, prophylactic treatment protocols, and diverse MS mouse models such as PLP-RR EAE, MOG-induced EAE, CPZ-induced demyelination, and acute lysolecithin demyelinating models. These factors complicate direct comparisons and interpretations of ligand effects on remyelination and neuroprotection. Notably, IndCl and its analogues, have shown significant remyelination and neuroprotection, and immunomodulatory effects, in mouse models of MS^11,14,15,23^. These findings suggest that certain structural modifications of ERβ ligands can enhance their therapeutic potential. Optimizing ERβ ligands for therapeutic use in MS and other neuroinflammatory conditions necessitates a nuanced understanding of their pathway selectivity and functional specificity. Relying solely on receptor binding affinity is insufficient; the ability of the ligand in the ERβ-ligand complex to engage specific signaling cascades and recruit appropriate cofactors that modulate its functions are crucial determinants of its biological efficacy.

*The development of chloroindazoles and their prospects as ERβ-selective MS therapeutics.* Building upon these findings, we have developed novel ERβ-selective chloroindazole analogues, K102 and K110, with the aim of advancing them toward IND status for MS clinical trials. Structure–activity relationship studies of over 60 related compounds were conducted, evaluating progressively their ERα and ERβ binding affinities, ADME and pharmacokinetic properties, and their OL differentiation potential in primary mouse cultures. K102 and K110 emerged as lead candidates, demonstrating superior remyelinating activity in both the CPZ and the EAE models. Neither compound showed significant potential to inhibit cytochrome P450-mediated metabolism, and both compounds demonstrated significant oral bioavailability with adequate pharmacokinetic results across the four species tested. A study of K_p uu_ in brain confirmed that K102 and K110 cross the blood–brain barrier and result in favorable brain to plasma ratios for total and unbound ligand. The same study showed high uptake in the uterus, especially with K110, but the lack of elevation of uterine weight confirms the lack of appreciable action through ERα, minimizing concerns related to ERα-mediated side effects.

In EAE models, therapeutic treatment with K102 and K110 during peak disease resulted in significant reductions in clinical scores, improved motor performance, and increased numbers of mature OLs and myelinated axons , decreased numbers of immune cells in the ON, and modulated peripheral immune responses. Furthermore, K102 treatment significantly alleviated EAE-induced RNFL thinning and improved VEP and ERG amplitudes, suggesting neuroprotective effects in the visual pathway. Notably, these are disease parameters that can be evaluated non-invasively as responses during a clinical trial.

K102 treatment selectively modulates transcriptional programs relevant to remyelination and mitochondrial health in chronic EAE. While EAE optic nerves showed widespread dysregulation of genes involved in myelination, inflammation, and axonal function^28^, K102 altered only a limited subset, including *Ccr2* and *Cpt1b,* suggesting targeted effects on immune recruitment and metabolic pathways. Notably, K102 restored expression of *Sox10* and *Olig2*, key regulators of OL differentiation, consistent with a pro-remyelinating effect. In contrast, complement-related inflammatory genes *(C1qa, C1qb, C1qc, C3*) and other immune markers remained unaffected, indicating that K102 does not broadly suppress neuroinflammation. In axonal pathways, K102 enhanced *Arc* and *Pvalb,* genes linked to synaptic plasticity and GABAergic activity, suggesting potential role in neuronal connectivity. The most pronounced effects were observed in mitochondrial genes: *Pink1, Tfam, Mfn2, Park7*, and *Sirt2* which were restored to near normal levels, supporting improved mitochondrial biogenesis and stress resilience in diseased tissue. Together, these results indicate that K102 facilitates axon remyelination, enhances neuronal support, and strengthens mitochondrial function, while exerting limited effects on complement-mediated inflammation. This profile highlights K102 as a candidate therapy that acts through repair and metabolic resilience rather than broad immunosuppression.

In conclusion, the development of ERβ-selective chloroindazoles, particularly K102 and K110, offers a novel therapeutic approach for promoting remyelination and immunomodulatory neuroprotection in MS. These compounds have demonstrated significant efficacy in preclinical models, addressing the unmet need for dual therapeutic actions to repair myelin and preserve neuronal function. Advancing these candidates into clinical trials holds the potential to transform the treatment landscape for patients with MS.

## Supplementary Information

Below is the link to the electronic supplementary material.

Supplementary Material 1

Supplementary Material 2

## Data Availability

All data generated or analyzed during this study are included in this published article [and its supplementary information files].
