# Supplementary material for: Chloroindazole based estrogen receptor β ligands with favorable pharmacokinetics promote functional remyelination and visual recovery
Source: Sci Rep. 2025 Oct 8;15:35056. doi: 10.1038/s41598-025-20254-9 (PMC12508063; doi:10.1038/s41598-025-20254-9)
Supplement: Supplementary file 1 — Supplementary Material 1 [file 41598_2025_20254_MOESM1_ESM.docx]

**Supplementary Materials and Figures**

**Feri et al 2025**

**Supplementary Methods**

*Experimental Animals:* All procedures were conducted according to the National Institutes of Health guidelines and approved by the Institutional Care and Use of Laboratory Animals Committee (IACUC) at the University of California, Riverside. Except where noted otherwise, wildtype C57BL/6J mice were used for these experiments. Mice were bred and housed at UCR vivarium facilities. Mice were kept on a 12‐hour light/dark cycle with unrestricted access to food and water.

*PKA Methods:* Pharmacokinetic studies were conducted by Pharmaron Ltd. using CD1 mice at doses of 5 mg/kg for subcutaneous (SC), 10 mg/kg for intravenous (IV), and 25 mg/kg for oral (PO) administration, and by Pharmaron using four female species: mice, rats, beagle dogs and cynomolgus monkeys and dosing in each case three animals at 40 mg/kg PO.

*K_p uu brain_* and K_p uu uterus_ Study: Female mice were treated with a single oral dose of 40 mg/kg **K102** and **K110** and samples from plasma, brain, and uterus were collected from three individual mice at four time points (0.5, 2, 4, and 8 hours). Plasma samples were 100% plasma or 5-fold diluted plasma; brain homogenate samples were diluted 5-fold with buffer, while uterus homogenate samples were initially diluted 5-fold and then further diluted to 20-fold. Equilibrium dialysis was performed in a 96-well plate system with wells containing 80 µL of buffer and 80 µL of the sample on opposite sides of the membrane. After incubation at 37°C for 5 hours with shaking at 180 rpm, aliquots were collected from both sides of the membrane for further analysis.

For plasma samples, 50 µL of sample was mixed with 50 µL of phosphate buffer, followed by addition of 300 µL quenching solution containing internal standards and DMSO. For 5-fold diluted plasma samples, 35 µL of sample was mixed with 35 µL of blank buffer, followed by 280 µL quenching solution. For tissue homogenate samples, 50 µL samples were mixed with 50 µL of phosphate buffer, followed by 200 µL quenching solution. All samples were vortexed for 1 minute, and centrifuged at 4,000 rpm for 15 minutes, and the supernatant collected for LC-MS/MS analysis, which was performed using an API 5500 mass spectrometer with an ACQUITY UPLC BEH 1.7 μm C18 100 Å column (2.1 mm * 50 mm) and ACE Excel 5 C4 (2.1 * 50 mm) column. Mobile phases were 0.1% formic acid in water or 5 mM NH_4_OAc with 0.05% formic acid in water (A) and 0.1% formic acid in acetonitrile (B). Run times were 8 minutes for plasma samples and 5 minutes for other sample types. The mass spectrometer was operated in positive electrospray ionization mode. Multiple reaction monitoring (MRM) was used for **K102** detection, with the transition 275.100 / 240.000 and 277.21 / 242.10 for **K110** detection.

Free fraction (fu) and recovery were calculated using specific equations for each sample type. For plasma samples, fu (%) was calculated as (Cbuffer / Cplasma) * 100%, and recovery (%) as ((Cplasma + Cbuffer) / T0) * 100%. For 5-fold diluted plasma samples, fu,20% (%) was calculated as (Cbuffer / Cplasma) * 100%, and fu,100% (%) as fu,20% / (5 - 4 * fu,20%) * 100%. Similar equations were used for uterus and brain samples, accounting for their respective dilution factors. These calculations allowed for the determination of the free fraction of the test compound in various tissue compartments and the assessment of recovery rates. The unbound concentration of **K102** or **K110** in brain or uterus compared to their unbound concentration in plasma (i.e., K_p uu_) was determined.

*Primary OL Progenitor cells (OPC) and Neuronal Cultures:* OPC and cortical neuronal cultures were prepared from the cortices of postnatal day P0-P1 C57BL/6J mice of both sexes, following established protocols (1, 2);(3). The single-cell suspension was divided into two tubes. One tube was treated with warm Neuronal Basal media, counted, and plated onto poly-L-lysine (PLL)-coated glass coverslips. The second tube was resuspended in glial growth medium, composed of DMEM-F12 supplemented with 10% fetal bovine serum, and placed in a PLL-coated culture flask for 8–10 days until confluent. Primary OPCs were then isolated by shaking and either co-cultured with primary neurons or plated onto poly-D-lysine-coated glass coverslips (three coverslips per condition, 2x10^6^ cells per coverslip). The OPCs were allowed to attach for three days, followed by five days in differentiating media (DMEM-F12 with Sato media containing triiodothyronine and thyroxine) supplemented with various analogues and remyelinating compounds. For comparison, a negative control (vehicle, consisting of the media and ethanol mixture used to dissolve the analogues and remyelinating compounds) and a normal control (differentiating media alone) were included in the experiments.

*Myelinating cocultures of primary Neuronal +OPC:* Dissociated brain cells were suspended in serum-free Neurobasal medium supplemented with 2% B27 (Gibco, Carlsbad, CA, USA) (Franco et al., 2015) and seeded onto poly-L-lysine-coated coverslips. The medium was refreshed every third day. After 12 days in culture, OPCs were isolated from mixed glial flasks by shaking, counted, and added to each well at a density of 5,000 cells per coverslip. Following a 2-hour incubation to allow cell attachment, the medium was aspirated and replaced with a 1:1 mixture of Neuronal Basal Media and Proliferation Media, totaling 1 mL per coverslip well. The neuronal/OPC co-culture was maintained for 3 days. After this period, the medium was switched from the NB/Proliferation Media mixture to NB/Maturation Media containing various test compounds, vehicle controls, or naïve media controls. The cultures were then maintained for an additional 5 days, with the medium replaced every 2 days.

*Human IPSC derived OL Culture and Treatment:* To assess the effects of analogues on the differentiation capacity of human OLs, Tempo’s iOligo™ cells were expanded in Tempo-iOligo™ growth medium, split and stored under liquid nitrogen. Vials were removed and brought to room temperature. Cells were counted and plated on Matrigel coated coverslips at a concentration of 12,000 cells/coverslip. After 3 days in growth media, it was replaced with Tempo-iOligo™ maturation medium+T3 (200ng/ml) containing vehicle and remyelinating drugs at the given concentrations for 3 weeks to induce differentiation. OLs were characterized using O4 and MBP IHC were used to assess the growth and differentiation to myelin producing cells.

*Quantification of Mature OLs and Co-culture axon myelination:* At the conclusion of the treatment period, cells were fixed and subjected to immunocytochemistry to evaluate myelin basic protein (MBP) expression, co-stained with the nuclear marker DAPI (shown in blue). The coverslips were imaged using an Olympus BX61 confocal microscope (Olympus America Inc., Center Valley, PA) at 10X magnification, with three images captured per coverslip. Additional high-resolution images were taken at 40X magnification. Cell counting was performed using the ImageJ multipoint tool, and cell densities were calculated by dividing the counts by the image area (mm²). The average cell density for each condition was normalized to the cell density of the normal control condition.

To assess OL differentiation, the number of MBP-positive (MBP+) cells and process extensions exceeding the diameter of the cell body were quantified. Additionally, highly branched MBP+ cells (with three or more processes) were counted (references). Co-culture images were processed and analyzed for β3-tubulin and MBP fluorescence using JACoP, an ImageJ toolbox for subcellular colocalization analysis. JACoP integrates global statistical methods and an object-based approach (4), and results were validated through manual quantification of myelinated axons. Data was analyzed and visualized using Prism software.

*EAE induction and treatment:* Eight to ten-week-old wild-type C57BL/6J mice were induced with active EAE using MOG_35-55_ peptide as previously described (1-5). Briefly, Pertussis toxin (PTX, Hooke Labs, BT-0105, Lot#: 1015) was administered on Day 0 and Day 2. Animals were scored daily for clinical EAE scores as follows: 0, unaffected; 1, complete tail limpness; 2, failure to right upon attempt to roll over; 3, partial hind limb paralysis; 4, complete hind limb paralysis; and 5. At peak disease 18-22 dpi, mice were scored and separated into 3 groups: EAE+V, EAE+SHK-K102, and EAE+K110. Analogs at different concentrations were dissolved in vehicle (10% ethanol and 90% miglyol 812N) at a dosage of 5mg/kg/day. At peak disease, average mouse body weights were used to calculate drug dosage. Mice received daily subcutaneous injections of drug or vehicle starting at peak disease. Four different EAE experiments are shown in Figure 4ii.

*Rotarod Motor Performance:* Mice cages were moved to the behavioral room 1 hour prior to testing. Motor performance was assessed using the Rotarod apparatus (Med Associates, Inc., St. Albans, VT) as previously performed with modifications(5, 6). Briefly, mice were placed on the rotating horizontal rod set to a speed of 3 – 30 rpm for a maximum of 300 seconds. The amount of time the mouse was able to walk on the accelerating rod was recorded. Each mouse was tested in three trials separated by 15 minute intertrial intervals. The average of the three trials was reported as a single value for each mouse. Statistical analysis was performed in GraphPad Prism 10 software using Mann-Whitney test.

*Cuprizone administration and treatment:* Mice were fed a diet containing 0.2% bis(cyclohexanone) oxaldihydrazone (cuprizone/CPZ, Teklad Custom Diets, Madison, WI) for 12 weeks, as previously described (7, 8) A subset of mice was perfused immediately after the 12-week cuprizone regimen (12wkDM). After the 12-week cuprizone treatment period, the other mice were switched back to a normal diet and treated with either vehicle (3wkRM+V), SHK-**K102** (3wkRM+**K102**), or **K110** (3wkRM+**K110**) for 3 weeks.

*Optical coherence tomography:* OCT was acquired using the spectral domain‐OCT (R2200 840 nm HHP; Leica, Deerfield, IL) at peak EAE disease according to previously published methods(8, 9). Each image was taken 3 times and averaged. Automatic segmentation of retinal layers was performed using Bioptigen Diver 3.0 software (Leica Microsystems, Deerfield, IL).

*Electroretinograms and visual evoked potentials* were measured using the HMsERG200 (OcuScience, Henderson, NV) as previously described (8, 9). Animals were dark adapted for a minimum of 5 hours prior to recording. During acquisition, animals were anesthetized with continuous 2% isoflurane. Traces were averaged and filtered for 60 Hz noise and a low-pass filter. MATLAB was used to adjust baselines and smoothed prior to measuring ERG and VEP amplitudes and latencies.

*Perfusions, tissue preparation, and IHC:* Mice were deeply anesthetized with isoflurane and intracardially perfused with ice‐cold PBS followed by 10% formalin in PBS (Fisher Scientific, Hampton, NH). Eyes, optic nerves, and brain were collected and processed for IHC as previously described(8, 9). Antibody details are listed in **Supplementary Table S6**.

*Splenocyte Isolation and Cytokine Analysis:* Spleens were removed from mice prior to transcardial perfusion. After isolation, splenocytes were counted and resuspended in RPMI. Following incubation, splenocytes were stimulated with 25 µg/ml MOG_35–55_ and supernatants were collected 48 hours later. Levels of the following cytokines and chemokines present in culture supernatants were sent to The Cytokine Core (Indianapolis, IN); pro-inflammatory cytokines: IFNγ, Tumor necrosis factor (TNF)α, IL-1α, IL-1β, IL-3, IL-6, IL9, IL17, Vascular endothelial growth factor (VEGF), CCL5/RANTES and macrophage colony-stimulating factor (M-CSF); anti-inflammatory cytokines: IL-5 and IL-10; chemokines: CXCL10, and CXCL1 were determined.

*Imaging and Quantification:* Retina, optic nerve, and brain sections were imaged using an Olympus BX61 spinning disk confocal microscope equipped with 10x and 40x Super Apochromat objectives (Olympus America Inc., Cypress, CA) connected to a camera (Hamamatsu Orca‐R2). Z‐stack images were acquired, and projection images were compiled using Slidebook 6 and cellSense software (Intelligent Imaging Innovations Inc, Santa Monica, CA). Immunofluorescence intensity and cell numbers were assessed with NIH ImageJ software (v1. 50i http://rsb.info.nih.gov/ij/) and quantified. Results from all counts were analyzed in GraphPad Prism for statistical significance.

*RNA Extraction and NanoString nCounter Gene Expression Assay:* RNA was isolated from a single optic nerve as described previously in(9). Optic nerves were isolated from normal, EAE+V, and EAE+K102 mice. RNA isolates were run on the nCounter Mouse Neuropathology panel (NanoString Technologies, Seattle, WA). Normalized data was used to calculate fold change for all three groups.

*Statistics:* All analyses were performed using GraphPad Prism 6 (La Jolla, CA). For cell culture experiments, each condition was tested in triplicate. Data were analyzed by one-way ANOVA to compare mean values across three or more groups, followed by Tukey’s post hoc test for multiple comparisons. Results are reported as mean ± SEM, with statistical significance set at α ≤ 0.05. For EAE studies, disease complexity was accounted for by including variables such as time, disease severity (clinical scores), and treatment. Because group sizes were unequal due to disease-related mortality, EAE scores were analyzed using a two-way ANOVA for unbalanced data, followed by Dunnett’s multiple comparisons test (as described in Hasselmann et al., 2017).

For IHC, data were obtained from three sections per mouse per region of interest (brain, optic nerve, retina), averaged to generate a single data point. Each group included 4–6 mice.For ERG, VEP, and OCT analyses, data from both eyes were included, with 6–8 mice per treatment group. Comparisons among groups (NM vs. 12wkCPZ (DM), 12wkDM vs. RM+V, RM+V vs. RM+K102, and RM+V vs. RM+K110) were performed in batches with a combination of vehicle treated + either K102 (or K110)-treated animals. As we were not able to record all the animals at the same time, we compared them using one-way ANOVA followed by Fisher’s LSD test.

For nCounter gene expression analysis, normalized counts were used to calculate log2 fold changes. Welch’s t-test (unequal variance t-test) was applied when comparing NM vs. EAE+V and EAE+V vs. EAE+K102, as both sample sizes and variances differed between groups. Significance levels were set as follows: *p < 0.05, **p < 0.01, ***p < 0.001, and ****p < 0.0001. Summary of statistics for Figures is in a table format in the supplementary sections Supplementary TableS7-S12 Significance levels were set as follows: *p < 0.05, **p < 0.01, ***p < 0.001, and ****p < 0.0001. Summary of statistics for Figures is in a table format in the supplementary sections **Supplementary TableS7-S12**.

**Supplementary Figures:**

**Figure S1. Reporter gene activation assay of K101, 102, 110, other known ERβ selective compounds, ERα selective PPT, and non-selective estradiol and DES.**

**Figure S2. Pharmacokinetic Behavior of K102 in mice. (A)** Mice were dosed with **K102** IV (5 mpk), SC (10 mpk) and PO (25 mpk) in three CD1 mice and levels of unmetabolized **K102** in plasma and brain were determined by LCMS out to 24 h. Only values above the LLOQ.(0.3 ng/mL) were plotted. **(B)** Plot of the ratio of **K102** in brain/plasma are plotted. For summary, see **Table S3;** for details, see Methods.

**
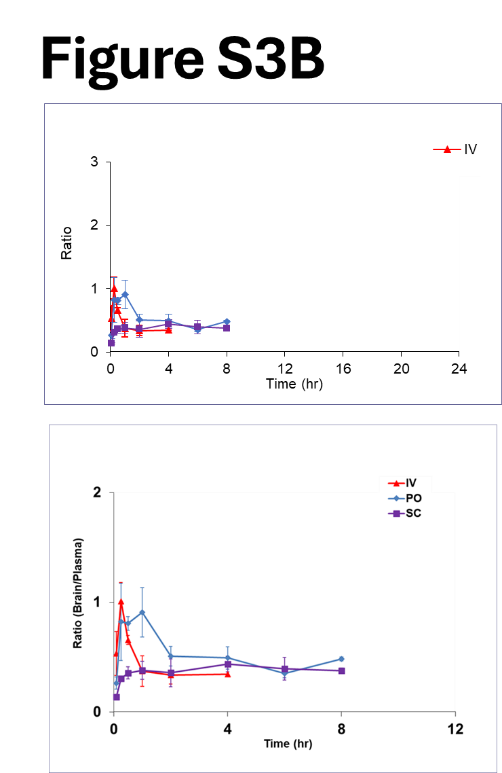
**
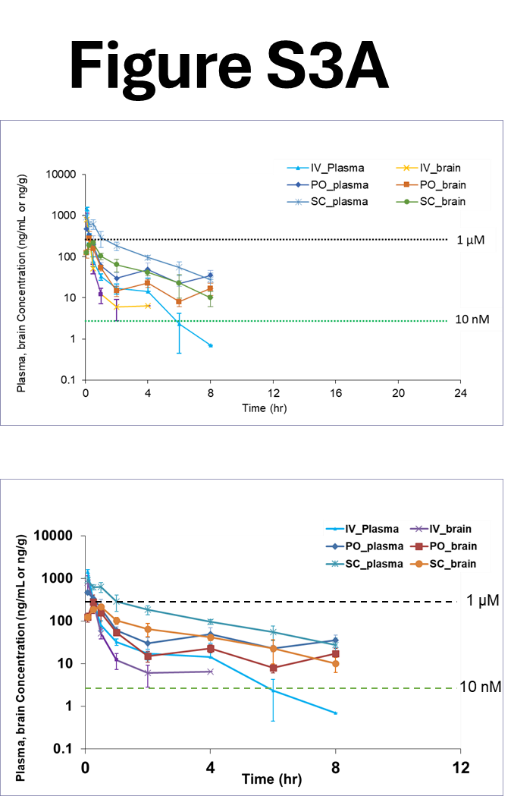
**Figure S2A** **Figure S2B**

**Figure S3. Pharmacokinetic Behavior of K110 in mice. (A)** Mice were dosed with **K110** IV (5 mpk), SC (10 mpk) and PO (25 mpk) in three CD1 mice and levels of unmetabolized **K110** in plasma and brain were determined by LCMS out to 24 h. Only values above the LLOQ.(0.3 ng/mL) were plotted. **(B)** Plot of the ratio of **K110** in brain/plasma are plotted. For summary, see **Table ;** for details, see Methods.


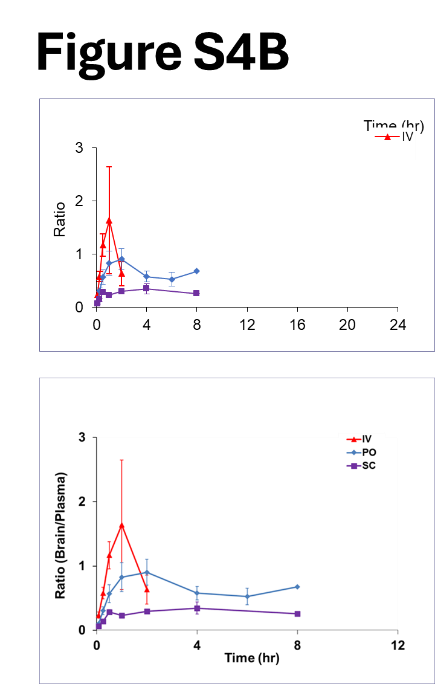

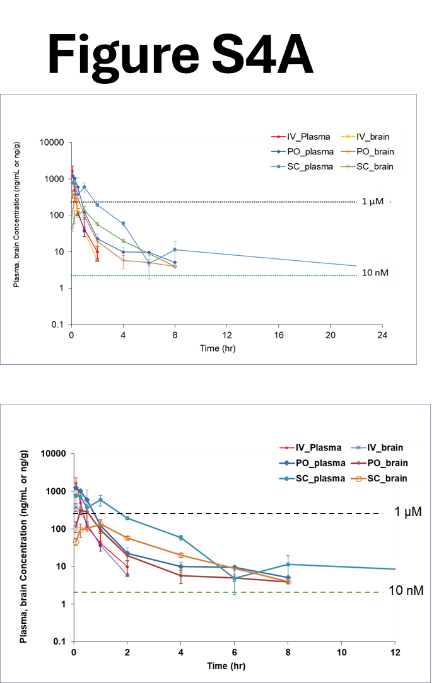
**Figure S3A Figure S3B**

**Figure S4. PK graph of blood levels for K102 administered orally at 40 mg/kg to female mice, rats, beagle dogs, and cynomolgus monkeys**

**
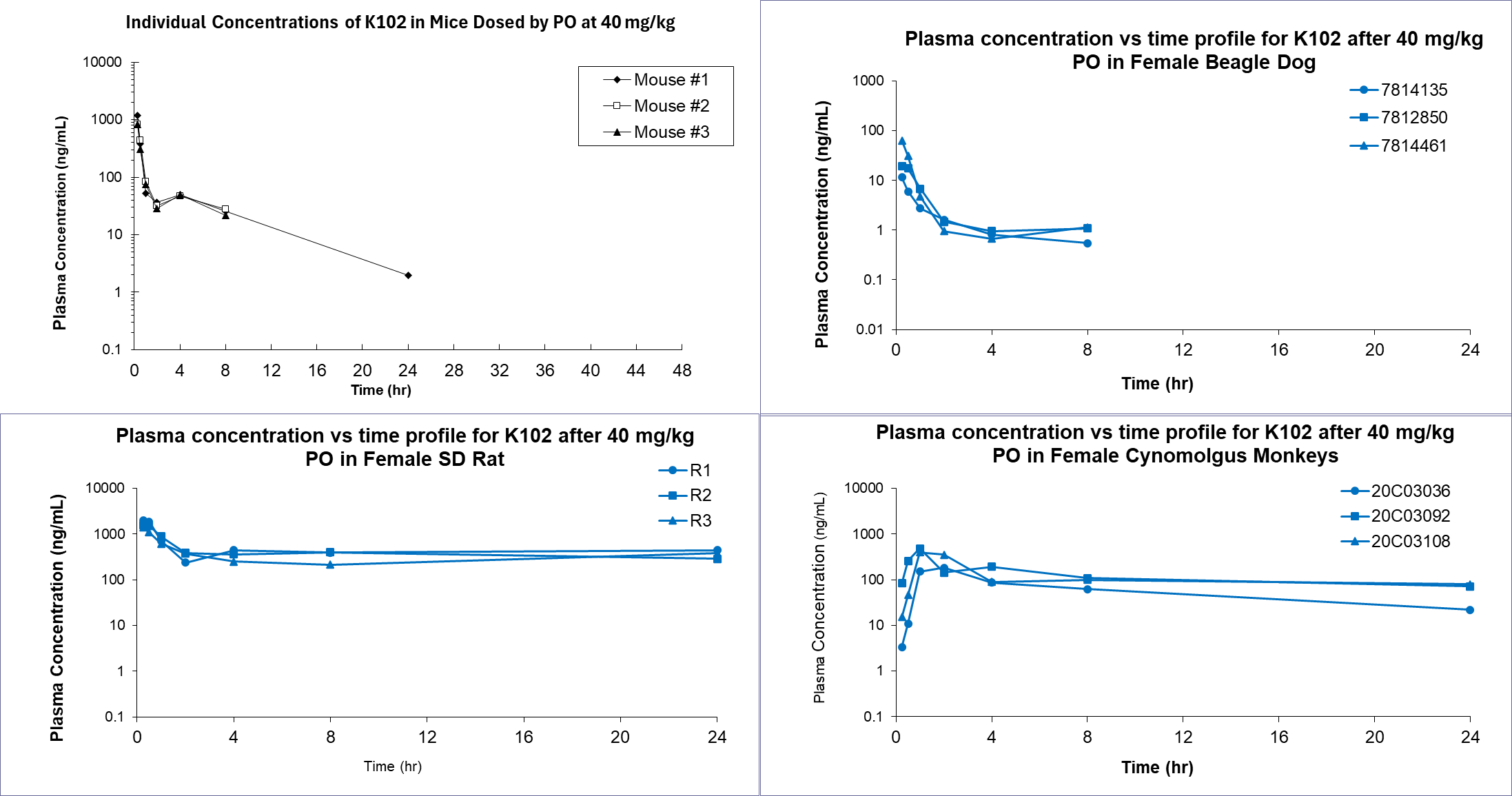
**

**Figure S5. OL differentiation in response to different doses of each compound. (S5i-ii)** The extent of OL differentiation was assessed in the presence of two doses (10nM and 100nM) of **K101**, **K102**, and **K110**. **(S5iii)** The total number of cells did not change, however, a significant increase in MBP+ OLs were observed compared to vehicle. Summary of all results can be found in **Table S7**. All graphs represent mean + SEM. *p<0.05, **p<0.01, ***p<0.001, ****p<0.0001 level.


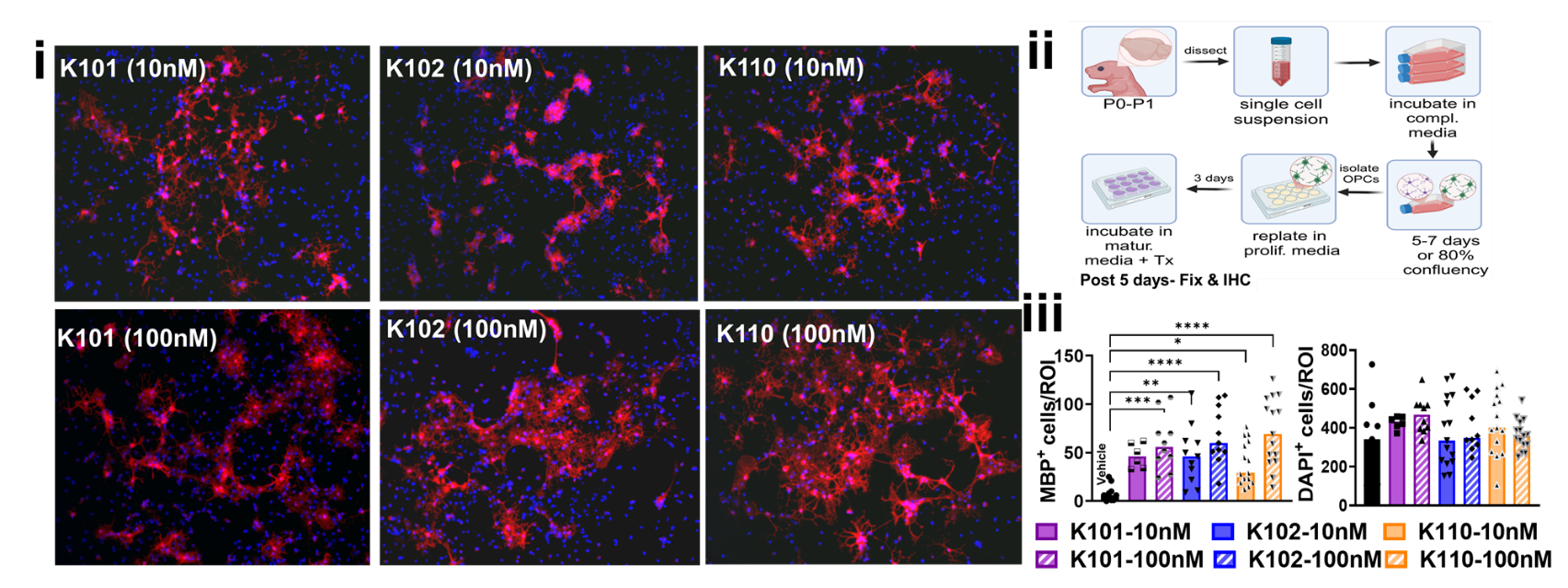


**Figure S6. Additional results from the EAE experiment illustrated in Figure 4Bii. (S6i)** Evaluation of disease severity recovery by behavior on a rotarod. The animals from Hook EAE experiment Figure 3Bii were used. In the past we have performed rotarod on our 2-MOG EAE mice and seen significant improvements with K102 (IndCl-o-Me)(2).  **(S6ii)** The weight of the uteri removed from mice relative to total body weight along with the raw uterine weight and body weight at the end of this experiment is plotted.

**
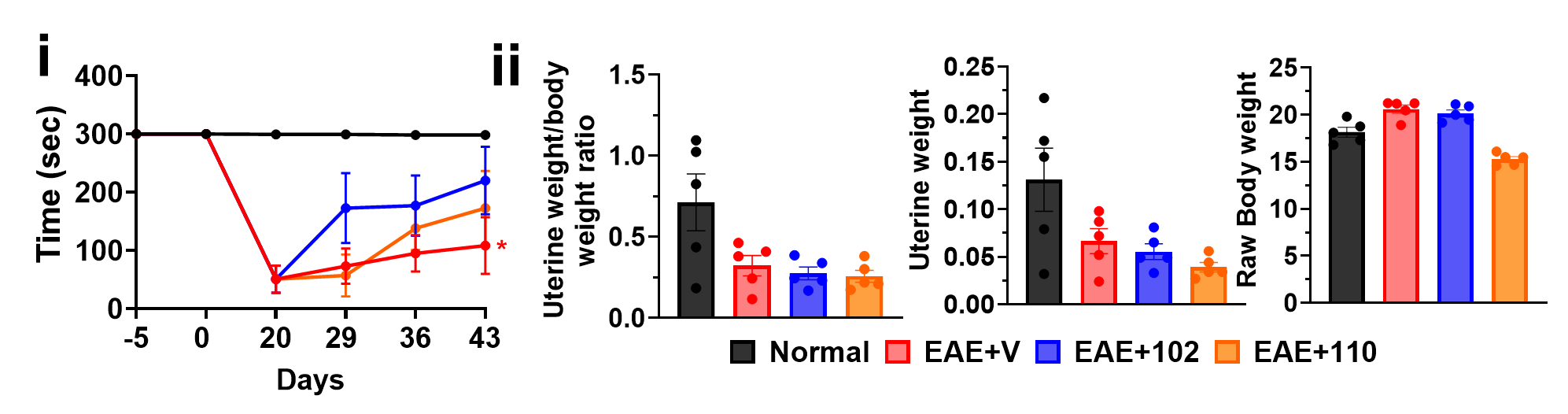
**

**Figure S7. Additional IHC results of the optic tract from EAE experiment illustrated in Figure 4Bi. (S7A)** Representative images of brain sections containing the optic tract are shown for NM, EAE+V, and EAE+K102 groups. **(S7Ai,iv)** Myelination, **(S7Aii,v)** microglia/ macrophage activation and **(S6Aiii,vi)** astrogliosis was evaluated. Treatment with **K102** improved myelination, and reduced microglia/macrophage activation and astrogliosis compared to vehicle. **(S7Bi)** Axon health and SARM1 **(S7Bii)** expression was evaluated in the optic tract. **K102** treatment improved axon health **(S7Biii)** and did not change SARM1 **(S7Biv)** expression when compared to vehicle. Summary of all results can be found in **Table S12**. All graphs represent mean + SEM. *p<0.05, **p<0.01, ***p<0.001, ****p<0.0001 level.

**
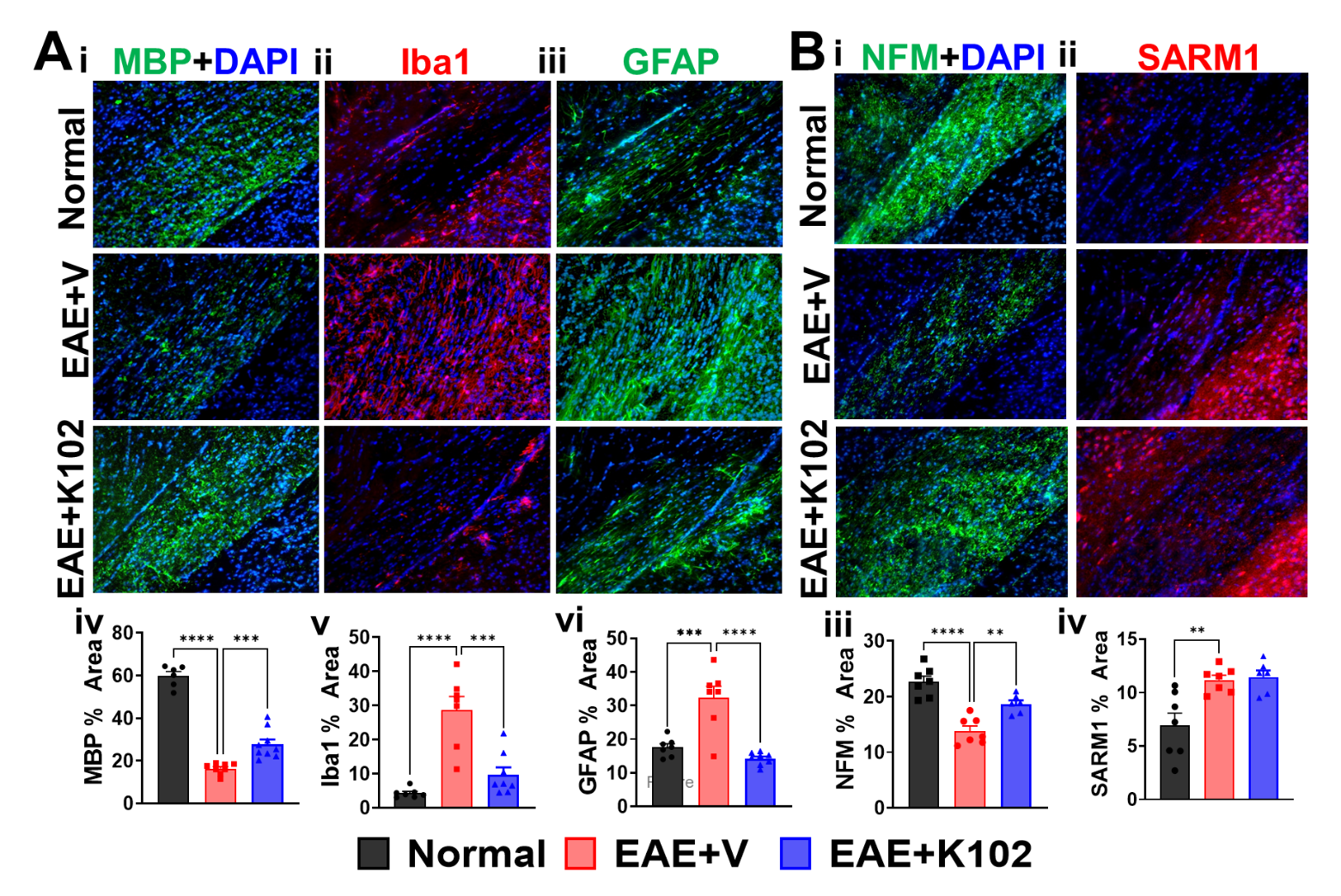
**

**Figure S8. Growth of human OLs in Matrigel vs. PLO coated coverslips.**

**(S8A)** Representative light microscope images at 10X of Tempo’s iOligo OPCs/OLs from wells treated with vehicle, K102, and K110 in Matrigel and PLO coated coverslips. Human OLs grew substantially better in the Matrigel coated coverslips compared to PLO coated coverslips. Future experiments were conducted on Matrigel coated coverslips. **(S8B)** Example images of human OLs treated with V, **K102, K110,** Clemastine, and ERB-0141 on Matrigel coated coverslips.

**
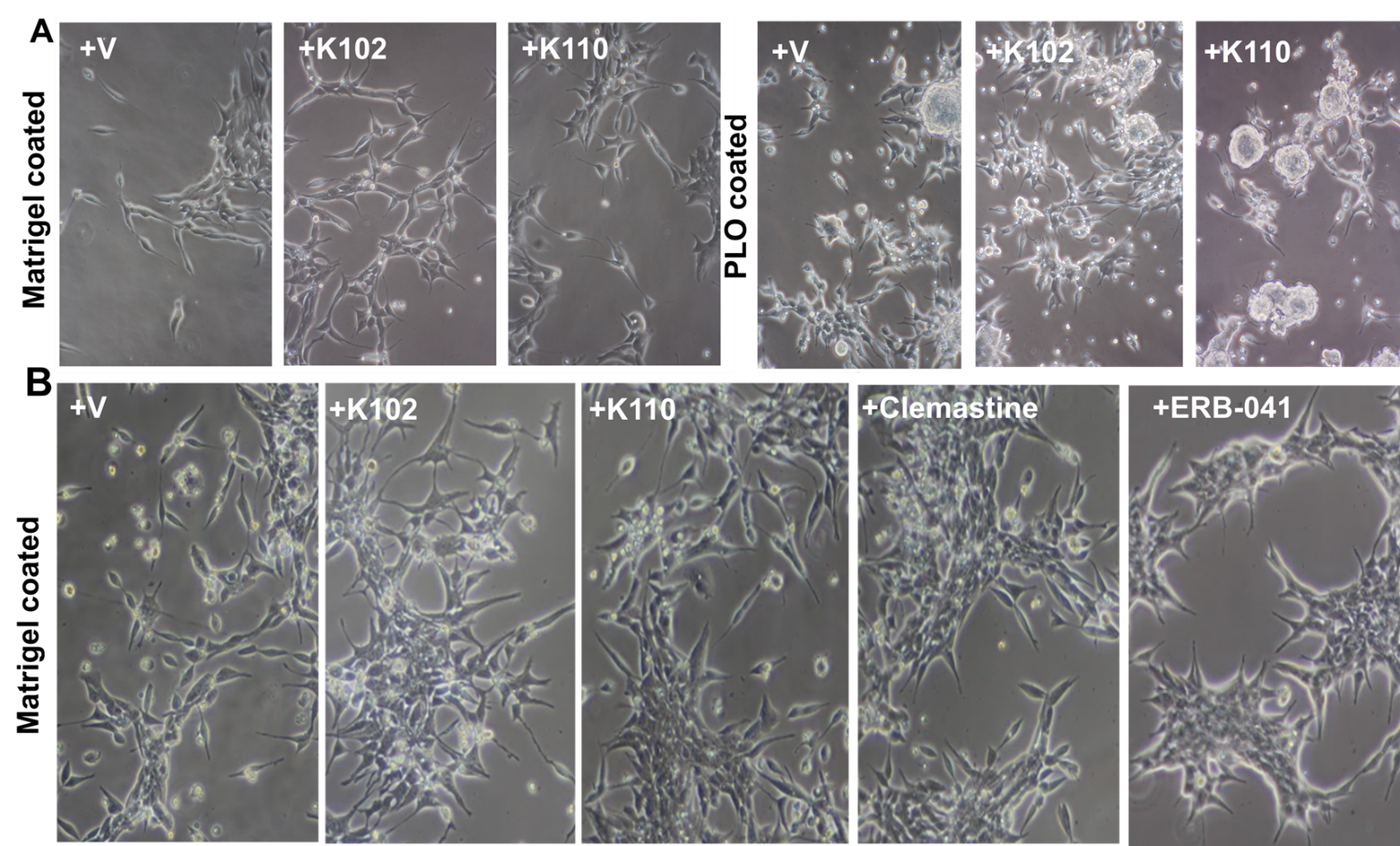
**

1. Sciarretta C, and Minichiello L. The preparation of primary cortical neuron cultures and a practical application using immunofluorescent cytochemistry. *Methods Mol Biol.* 2010;633:221-31.

2. Karim H, Kim SH, Lauderdale K, Lapato AS, Atkinson K, Yasui N, et al. Analogues of ERβ ligand chloroindazole exert immunomodulatory and remyelinating effects in a mouse model of multiple sclerosis. *Scientific Reports.* 2019;9(1):1-17.

3. Tiwari-Woodruff S, Beltran-Parrazal L, Charles A, Keck T, Vu T, and Bronstein J. K+ channel KV3.1 associates with OSP/claudin-11 and regulates oligodendrocyte development. *Am J Physiol Cell Physiol.* 2006;291(4):C687-98.

4. Bolte S, and Cordelieres FP. A guided tour into subcellular colocalization analysis in light microscopy. *J Microsc.* 2006;224(Pt 3):213-32.

5. Kumar S, Patel R, Moore S, Crawford DK, Suwanna N, Mangiardi M, et al. Estrogen receptor β ligand therapy activates PI3K/Akt/mTOR signaling in oligodendrocytes and promotes remyelination in a mouse model of multiple sclerosis. *Neurobiol Dis.* 2013;56:131-44.

6. Tiwari-Woodruff S, Morales LBJ, Lee R, and Voskuhl RR. Differential neuroprotective and antiinflammatory effects of estrogen receptor (ER) α and ERβ ligand treatment. *Proceedings of the National Academy of Sciences.* 2007;104(37):14813-8.

7. Lapato AS, Szu JI, Hasselmann JPC, Khalaj AJ, Binder DK, and Tiwari-Woodruff SK. Chronic demyelination-induced seizures. *Neuroscience.* 2017;346:409-22.

8. Sekyi MT, Lauderdale K, Atkinson KC, Golestany B, Karim H, Feri M, et al. Alleviation of extensive visual pathway dysfunction by a remyelinating drug in a chronic mouse model of multiple sclerosis. *Brain Pathol.* 2021;31(2):312-32.

9. Sekyi MT, Feri M, Desfor S, Atkinson KC, Golestany B, Beltran F, et al. Demyelination and neurodegeneration early in experimental autoimmune encephalomyelitis contribute to functional deficits in the anterior visual pathway. *Sci Rep.* 2024;14(1):24048.
